# Defense Regulatory Network Associated with circRNA in Rice in Response to Brown Planthopper Infestation

**DOI:** 10.3390/plants13030373

**Published:** 2024-01-26

**Authors:** Hou-Hong Yang, Ya-Xuan Wang, Jing Xiao, Yi-Fan Jia, Fang Liu, Wei-Xia Wang, Qi Wei, Feng-Xiang Lai, Qiang Fu, Pin-Jun Wan

**Affiliations:** State Key Laboratory of Rice Biology and Breeding, China National Rice Research Institute, Hangzhou 311400, China; 82101221215@caas.cn (H.-H.Y.); wangyaxuan604@163.com (Y.-X.W.); xiaojing00004@163.com (J.X.); jiayifanf@163.com (Y.-F.J.); qlyj0331@163.com (F.L.); wangweixia@caas.cn (W.-X.W.); weiqi01@caas.cn (Q.W.); laifengxiang@caas.cn (F.-X.L.); fuqiang@caas.cn (Q.F.)

**Keywords:** circular RNAs (circRNAs), *Nilaparvata lugens*, IR56 rice, rice-BPH interaction

## Abstract

The brown planthopper (BPH), *Nilaparvata lugens* (Stål), a rice-specific pest, has risen to the top of the list of significant pathogens and insects in recent years. Host plant-mediated resistance is an efficient strategy for BPH control. Nonetheless, BPH resistance in rice cultivars has succumbed to the emergence of distinct virulent BPH populations. Circular RNAs (circRNAs) play a pivotal role in regulating plant–environment interactions; however, the mechanisms underlying their insect-resistant functions remain largely unexplored. In this study, we conducted an extensive genome-wide analysis using high-throughput sequencing to explore the response of rice circRNAs to BPH infestations. We identified a total of 186 circRNAs in IR56 rice across two distinct virulence groups: IR-IR56-BPH (referring to IR rice infested by IR56-BPH) and IR-TN1-BPH, along with a control group (IR-CK) without BPH infestation. Among them, 39 circRNAs were upregulated, and 43 circRNAs were downregulated in the comparison between IR-IR56-BPH and IR-CK. Furthermore, in comparison with IR-CK, 42 circRNAs exhibited upregulation in IR-TN1-BPH, while 42 circRNAs showed downregulation. The Gene Ontology and Kyoto Encyclopedia of Genes and Genomes enrichment analysis revealed that the targets of differentially expressed circRNAs were considerably enriched in a multitude of biological processes closely linked to the response to BPH infestations. Furthermore, we assessed a total of 20 randomly selected circRNAs along with their corresponding expression levels. Moreover, we validated the regulatory impact of circRNAs on miRNAs and mRNAs. These findings have led us to construct a conceptual model that circRNA is associated with the defense regulatory network in rice, which is likely facilitated by the mediation of their parental genes and competing endogenous RNA (ceRNA) networks. This model contributes to the understanding of several extensively studied processes in rice-BPH interactions.

## 1. Introduction

The brown planthopper (BPH), *Nilaparvata lugens* (Stål) (Hemiptera: Delphacidae), is a phloem-feeding insect of cultivated rice (*Oryza sativa* L.), thus causing wilting and ultimately resulting in significant yield losses [1,2]. While the application of various insecticides is the prevailing method used to manage BPH infestations, the overuse of these chemicals has led to a range of negative outcomes, encompassing insecticide resistance, the resurgence of insects, the reduction of natural predator populations, and other environmental risks. As a result, the identification and molecular breeding of rice germplasms that possess BPH-resistance genes (referred to as *Bph*/*bph* genes) are regarded as the optimal approach for effectively controlling and managing BPH [3]. Up to the present, a total of 45 BPH resistance loci have been documented across various rice cultivars and wild-rice species [4]. Amongst them, *bph2*/*Bph26* [5], *Bph3*/*Bph17* [6], *Bph6* [7], *Bph9* [8], *Bph14* [9], *Bph15* [10], *Bph18* [11], *bph29* [12], *Bph30* [13], *Bph32* [14], and *Bph40* [13] have been successfully identified through map-based cloning methods. Particularly noteworthy is the *Bph3* locus, initially identified in the Sri Lankan indica cultivar Rathu Heenati. This locus constitutes a cluster of three genes that encode plasma membrane-localized lectin receptor kinases (OsLecRK1-OsLecRK3) [6]. *Bph3* has exhibited resistance to four distinct BPH biotypes, which are classified based on their virulence towards specific BPH resistance genes [15]. Recently, a new virulent BPH population or strain, termed IR56-BPH, was established. This population effectively overcame *Bph3*-mediated resistance to IR56 rice (harboring *Bph3*) [16]. After more than 40 generations of force-feeding, IR56-BPH successfully bypassed the resistance of IR56 rice (assessed using the standard seed box screening technique, Grade 7), demonstrating a notably increased emergence rate. This development indicated the evolution of virulence in IR56-BPH against IR56 rice. Nonetheless, the intricate molecular mechanism governing the interaction between IR56-BPH and its host plant remains insufficiently understood. 

Plants have evolved intricate sensory mechanisms to detect biotic invasions and counteract the negative impacts on growth, yield, and survival [17]. They possess cell-surface immune receptors as well as intracellular immune receptors that are responsible for detecting signals from microbes and insects [18,19]. Upon sensing these signals, plants initiate early immune responses characterized by processes such as calcium influx, reactive oxygen species burst, and mitogen-activated protein kinase activation [19,20]. These initial responses subsequently trigger downstream transcriptional reprogramming of defense-related genes, including transcription factors and genes involved in hormone synthesis. This orchestrated cascade of events eventually leads to the establishment of late immune responses.

Non-coding RNAs (ncRNAs) consist of diverse classes of RNA transcripts that are not translated into proteins. ncRNAs can be categorized into housekeeping RNAs (transfer RNAs (tRNAs), ribosomal RNAs (rRNAs), small nuclear RNAs (snRNAs), and small nucleolar RNAs (snoRNAs)) and regulatory RNAs (piwi-interacting RNAs (piRNAs), small interfering RNAs (siRNAs), microRNAs (miRNAs), long non-coding RNAs (lncRNAs), and circular RNA (circRNAs)) [21]. Among the ncRNA classes, miRNAs and lncRNAs have received the most attention in plants [21]. In plants with BPH resistance mediated by *Bph3*, *Bph15*, or *Bph36*, numerous miRNAs/lncRNAs were differently expressed in response to the infestation of biotype 1 or TN1 population of BPH [20,22,23,24]. Out of these miRNAs, three miRNAs (miR156, miR159, and miR396) have been reported to regulate rice resistance to BPH. Specifically, miR156 regulates jasmonic acid (JA) and jasmonoyl-isoleucine (JA-Ile) biosynthesis through the “miR156-OsMPK3/6-OsWRKY70” module, thus negatively regulating BPH resistance [25]. OsmiR159 and OsmiR396 also negatively regulate BPH resistance through the “OsmiR159-OsGAMYBL2-GS3” [26] and “OsmiR396-OsGRF8-OsF3H-flavonoid” module [27], respectively. Recent study revealed that lncRNAs and circRNAs act as competitive endogenous RNAs (ceRNAs) to regulate BPH resistance in rice [28]. However, as far as our current understanding goes, there are no existing reports that delve into the role of circRNAs in a resistant rice variety’s response to BPH infestations of varying virulence levels. Hence, the present situation presents a valuable opportunity to investigate the participation of circRNAs and their subsequent roles in modulating defenses during interactions between rice and BPH.

In the current study, we have undertaken a comprehensive examination of circRNA profiles in resistant IR56 rice during separate infestations by the virulent IR56-BPH and the avirulent TN1-BPH. The interaction between IR56 rice and IR56-BPH (referred to as IR-IR56-BPH) is characterized as compatible, whereas the interaction between IR56 rice and TN1-BPH (referred to as IR-TN1-BPH) is deemed incompatible in nature [20]. By performing circRNA sequencing on three distinct libraries—IR-IR56-BPH, IR-TN1-BPH, and a control without BPH infestation as IR-CK, we aimed to identify circRNAs involved in the interplay between rice and BPH. Furthermore, the identification of differentially expressed (DE) circRNAs in IR-IR56-BPH and IR-TN1-BPH provided insights into their specific roles in the interactions between rice and BPH. To reinforce their contribution to IR56 rice’s defense, selected DE circRNAs and their targets were validated through qPCR analysis. Predictions of circRNA targets and their functional annotations using GO and KEGG enrichments shed light on the defense-modulating roles of certain DE circRNAs and their targets. In conclusion, building upon the findings of this study, we introduce a novel model that outlines the circRNA regulatory network in IR56 rice, proposing a regulatory mechanism linked to defense, phytohormones, and growth-regulating factors. This mechanism seeks to unveil the functional significance of circRNAs in the context of rice-BPH interactions.

## 2. Results

### 2.1. Identification of Circular RNAs in IR56 Rice

To systematically discern the involvement of circRNAs in rice-BPH interactions, we constructed three RNA libraries from rice stems (IR-IR56-BPH, IR-TN1-BPH, and IR-CK) after 24 h of BPH feeding. Genome-wide high-throughput RNA sequencing was executed using the Illumina HiSeq 2500 platform (Illumina, San Diego, CA, USA) on these libraries, each yielding over 90 million raw reads. The total raw read counts were 106,174,788 for IR-CK control, 95,701,674 for IR-IR56-BPH, and 95,646,838 for IR-TN1-BPH (Supplementary Table S1). After excluding adapters and low-quality reads, raw reads underwent filtration to eliminate poly A tails and incorrect adapters. Consequently, we obtained 101,459,300 clean reads for the IR-CK, 91,463,396 for IR-IR56-BPH, and 91,019,610 for IR-TN1-BPH (refer to Supplementary Table S1). Furthermore, the Q30 scores for the clean reads exceeded 91.0%, and the GC contents of the sequencing outputs ranged from 47.93% to 48.90% (Supplementary Table S1).

### 2.2. The Distribution and Characterization of circRNAs in Three Libraries

**Figure 1:**
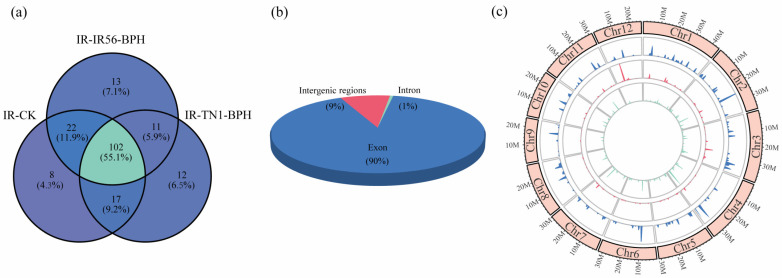
The distribution and characterization of circRNAs in three samples and circRNA validation. (**a**) Venn diagram showing the number and distribution of detected circRNAs in three libraries. (**b**) The number of circRNAs generated from exonic, intronic, and intergenic regions. (**c**) The density distribution (in 1 M window) of circRNA on chromosomes in all three libraries (inner to outer ring represent IR-CK, IR-TN1-BPH, and IR-IR56-BPH, respectively).

After a comprehensive screening and subsequent bioinformatic analysis, a total of 186 circRNAs were successfully identified across the three rice samples examined in this study (Figure 1). Of these, 102 circRNAs were consistently detected in all three rice samples (Figure 1a). The outcomes underscore that the expression of circRNAs is unique to the response against BPH infestations. These 186 identified circRNAs were collectively analyzed. Genomic origin analysis indicated that the majority of the identified circRNAs, specifically 847 (89.63%), were exonic circRNAs. A smaller portion, 15 (1.58%), represented intronic circRNAs, while the remaining 83 (8.79%) were categorized as intergenic circRNAs (Figure 1b). The examination of chromosome distribution revealed that circRNAs originated from each chromosome in rice (Figure 1c). The findings underscore the diverse genomic origins of rice circRNAs, with coding regions predominantly implicated.

For each individual sample, we carried out the quantification of circular RNA expression levels, followed by the normalization of these levels using the transcripts per million (TPM) metric. Circular RNAs surpassing 1000 TPM were categorized as abundantly expressed circRNAs, while those with TPM values below 10 were deemed rarely expressed circRNAs. The 20 most highly abundant circRNAs in each library are detailed in Supplementary Table S2. Expression level clustering analysis differential circRNA clustering analysis was used to determine the clustering pattern of differential circRNA expression under different experimental conditions. Clustering analysis is crucial in identifying patterns within a dataset, allowing us to group circRNAs based on their expression profiles. We applied circRNA cluster analysis to establish the clustering pattern of circRNA expression levels across diverse experimental conditions. Each comparison group yielded a distinct set of circRNAs, and the combined circRNA pools from all comparison groups were integrated into the TPM value of each experimental group. This was performed to facilitate hierarchical clustering analysis, K-means clustering analysis (illustrated in Supplementary Figure S1a), and self-organizing map (SOM) clustering analysis (see Supplementary Figure S1b). These findings offer valuable insights into the prospective functional roles of circRNAs by examining their relative expression levels under various experimental conditions.

### 2.3. Analysis of Differentially Expressed (DE) circRNAs Involved in the Response to BPH Infestations of Rice

**Figure 2:**
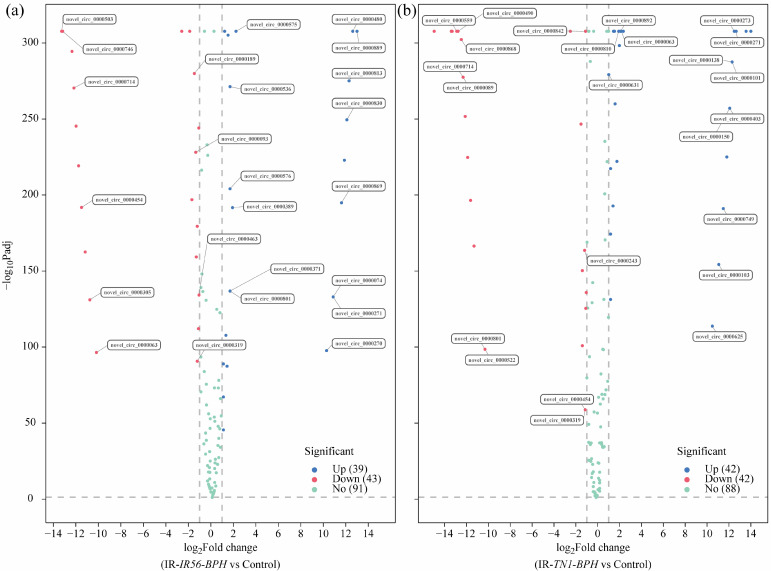
The volcano plots showing the differential expression of circRNAs in the comparisons between IR-IR56-BPH and IR-CK (**a**), as well as IR-TN1-BPH and IR-CK (**b**). The DE circRNAs exhibiting significant upregulation and downregulation are represented in red and green, respectively, with an adjusted *p* value < 0.01. The level of gene expression did not significantly differ between the two groups in the point of the blue condition (adjusted *p* value > 0.01).

The differential expression analysis of all identified circRNAs revealed that BPH infestations have significant effects on the transcript abundance of the IR56 rice circRNAs. Applying the screening criteria of fold change > 1 and adjusted *p* value (*q* Value) < 0.05, we identified differentially expressed (DE) circRNAs in two groups: IR-IR56-BPH versus IR-CK and IR-TN1-BPH versus IR-CK (Figure 2, Supplementary Table S3). The quantities of upregulated and downregulated DE circRNAs in each group were also presented (Figure 2). For instance, in the IR-IR56-BPH versus IR-CK group, there were 39 upregulated and 43 downregulated DE circRNAs (Figure 2a). Similarly, in the IR-TN1-BPH versus IR-CK group, 42 circRNAs exhibited upregulation, while 42 circRNAs displayed downregulation (Figure 2b).

Evidently, a discrepancy existed in the quantities of differentially expressed circRNAs between the two groups, suggesting a potential variation in circRNA responses to brown planthopper infestations of varying virulence levels. These DE circRNAs might fulfill distinct functions implicated in the rice-BPH interactions. The nature of the interaction, whether compatible or incompatible, influences the quantity, classification (family/novel), and regulation (up- or downregulation) of circRNA expressions in rice.

### 2.4. Functional Enrichment Analysis on DE circRNAs and Their Functional Annotations

**Figure 3:**
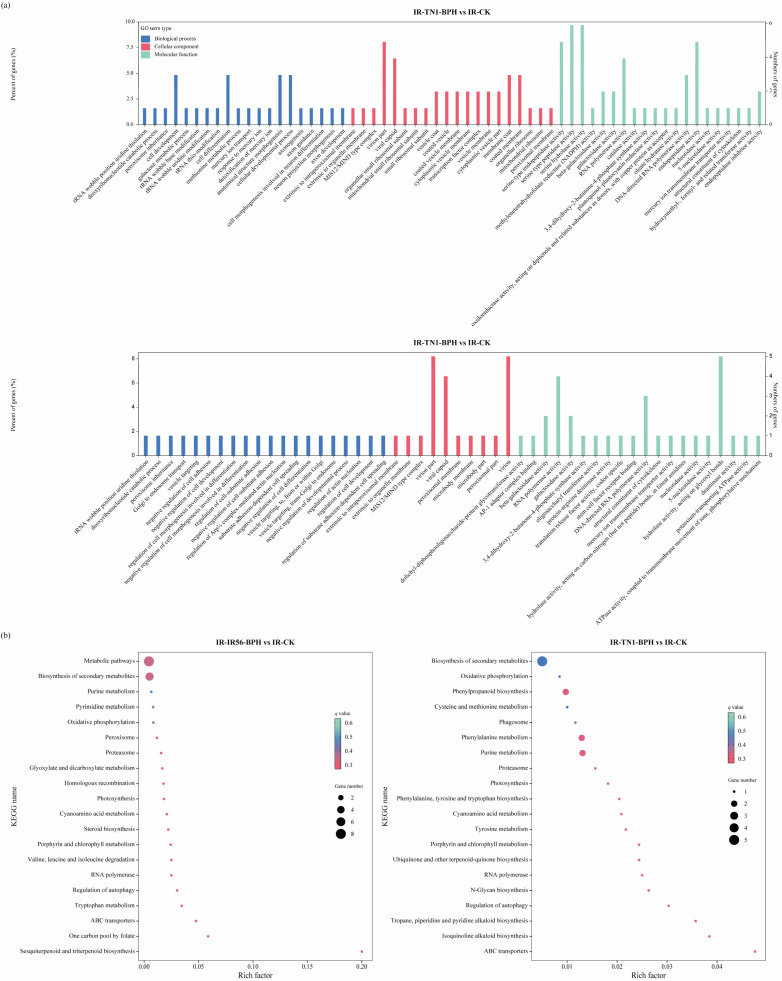
Gene ontology annotations (**a**) and top 20 KEGG pathways (**b**) enriched in the DE circRNA targets in IR-IR56-BPH and IR-TN1-BPH.

The identification of the target genes of the DE circRNAs provides an avenue to comprehend the potential regulatory functions of these circRNAs in defense modulation. For a deeper exploration of the potential roles of the identified DE circRNAs in the rice response to BPH infestations, functional enrichment analyses were conducted for circRNAs in both IR-IR56-BPH versus IR-CK and IR-TN1-BPH versus IR-CK comparisons. The results of DE circRNAs targets revealed that a single circRNAs in rice could have multiple miRNA targets, while a single miRNA transcript could also be targeted by multiple circRNAs. To functionally characterize the circRNA targets, Gene Ontology (GO) enrichment analysis and Kyoto Encyclopedia of Genes and Genomes (KEGG) pathway analyses were executed (Figure 3). The GO enrichment analysis demonstrated similar biological processes and molecular functions for the targets in both IR-IR56-BPH and IR-TN1-BPH comparisons, categorized under cellular component, molecular function, and biological process, respectively (Figure 3a).

Additionally, Kyoto Encyclopedia of Genes and Genomes (KEGG) pathway analysis was performed to delve further into the functionalities of circular RNAs. In the top 20 KEGG pathways of both IR-IR56-BPH and IR-TN1-BPH comparisons, 10 pathways were shared, while the remaining 10 were unique to each respective group. The KEGG pathway enrichments indicated that certain target genes of circRNAs from both IR-IR56-BPH and IR-TN1-BPH samples are commonly involved in physiological processes such as ABC transporters, biosynthesis of secondary metabolites, cyanoamino acid metabolism, and oxidative phosphorylation (Figure 3b).

However, the targets of IR-IR56-BPH and IR-TN1-BPH exhibited distinct and exclusive KEGG enrichments. For IR-IR56-BPH, these included glyoxylate and dicarboxylate metabolism, homologous recombination, metabolic pathways, and others, while IR-TN1-BPH featured enrichments in cysteine and methionine metabolism, isoquinoline alkaloid biosynthesis, N-glycan biosynthesis, phagosome, and more.

**Figure 4:**
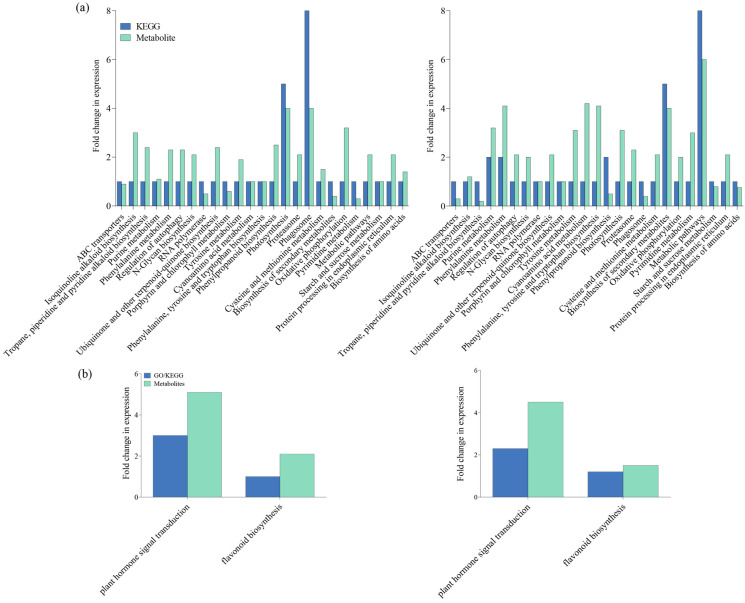
KEGG enrichment analysis of the DEGs (steelblue column) and DAMs (pale turquoise column) that were enriched in the pathway in IR-IR56-BPH and IR-TN1-BPH (**a**) and DEGs and DAMs mapped on plant hormone signal transduction and flavonoid biosynthesis (**b**).

To confirm our findings, we identified differently accumulated metabolites (DAMs) in both IR-IR56-BPH versus IR-CK and IR-TN1-BPH versus IR-CK comparisons. These included metabolic pathways such as cyanoamino acid metabolism, photosynthesis, and purine metabolism. Our results indicate substantial impacts on both secondary metabolite biosynthesis and overall metabolic pathways in IR-TN1-BPH versus IR-CK. Conversely, photosynthesis and phagosome pathways were notably affected in IR-IR56-BPH versus IR-CK (Figure 4a). Additionally, the analysis revealed that DAMs in both comparisons were significantly enriched in terms of plant hormone signal transduction and flavonoid biosynthesis (Figure 4b). Thus, the presence of shared enriched targets in metabolic pathways suggests commonalities in the BPH-feeding responses of IR56 rice, irrespective of BPH population. Nevertheless, the unique enhancements in circRNA targets hint at differing metabolic reactions in IR56 rice in response to BPH infestations based on the infesting population.

### 2.5. Display of the BPH-Feeding Response in IR56-Associated circRNAs and Their Putative Parental Genes and Validation of Selected DE miRNAs and Their Targets

**Figure 5:**
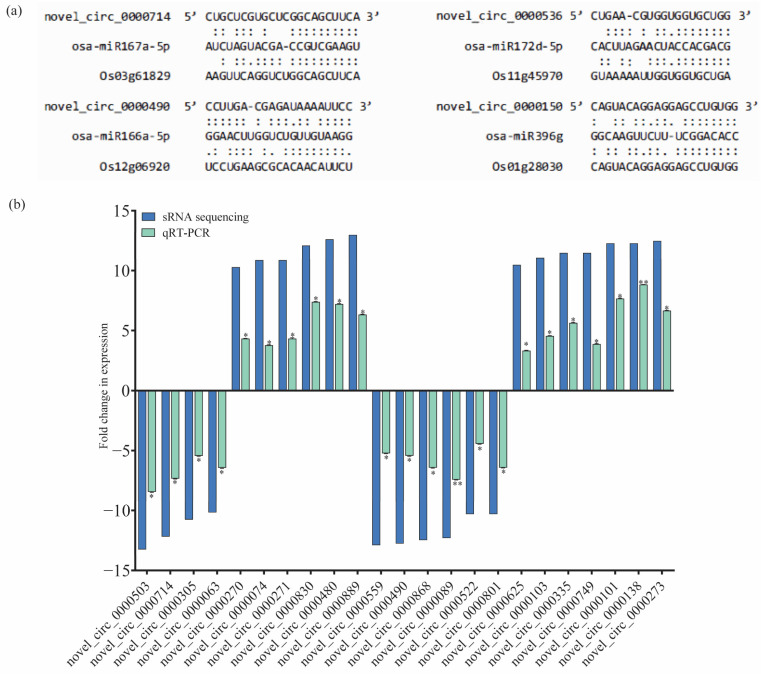
The circRNA-associated networks and their predicted mRNAs and expression validation of the selected miRNAs using qPCR. (**a**) The binding sites of circRNAs and miRNAs. (**b**) The fold changes (log2) in the expression of the circRNAs (qRT-PCR) were calculated and compared to the IR-CK group. Bars represent the mean ± SE of three biological replicates for the qPCR data. Asterisks * and ** indicate the significant difference in the expression levels of miRNAs in IR-IR56-BPH or IR-TN1-BPH as compared to IR-CKat *p* < 0.05 and *p* < 0.01, respectively (Student’s *t*-test).

In order to systematically investigate the potential regulatory roles of circRNAs in response to BPH feeding in IR56 rice, an analysis was conducted to identify gene sets associated with specific biological processes. Initially, protein-coding transcripts were identified through rRNA-depleted library RNA-seq, similar to the method used for circRNA identification. Subsequently, differentially expressed (DE) mRNA transcripts were obtained through pairwise comparisons. The TPM matrix of DE circRNA transcripts and the FPKM matrix of DE mRNA transcripts were then combined, along with specific information regarding predicted miRNA and circRNA binding sites (Supplementary Table S4). Illustrations depicting the interactions among circRNAs, miRNAs, and their corresponding target mRNAs are provided to visually represent the interactions for four selected circRNAs (Figure 5a).

To validate the transcript abundance of the identified circRNAs from small RNA sequencing, a subset of over 20 circRNAs was randomly chosen (from IR-IR56-BPH and IR-TN1-BPH 1). Their relative expressions were confirmed using real-time quantitative PCR (qPCR), with the primer details listed in Supplementary Table S6. The qPCR results aligned with the sRNA sequencing outcomes, demonstrating a consistent and significant trend of relative expression levels in the BPH-infested rice samples compared to the IR-CK (Figure 5b).

In our study, we utilized the IRESfinder software (v1.1.0) to detect the presence of internal ribosome entry site (IRES) elements within circRNA sequences. The index value in Table 1 represents the prediction outcome indicating the likelihood of IRES existence within the corresponding circRNA. This information provides insight into the level of confidence in the circRNA’s possession of IRES (a score closer to 1 indicates a higher degree of confidence in the presence of IRES within the circRNA). These findings suggest that circRNAs might potentially encode peptides, which holds significant implications for understanding the functional roles of circRNAs in plants. This discovery supports and lays the groundwork for further research to explore the functional aspects of circRNAs in rice.

### 2.6. Exploring the Potential Roles of BPH-Feeding Response in IR56-Associated circRNAs

**Figure 6:**
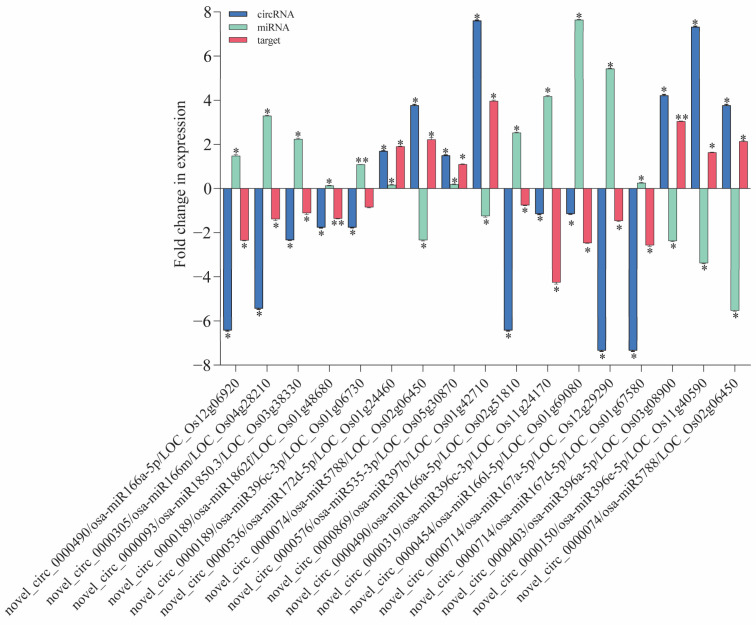
The qRT-PCR validation of DE circRNAs and their co-expressed and possibly regulated mRNAs in the ceRNA network. Bars represent the mean ± SE of three biological replicates for the qPCR data. Asterisks * and ** indicate the significant difference in the expression levels of ceRNAs in IR-IR56-BPH or IR-TN1-BPH as compared to the IR-CK group at *p* < 0.05 and *p* < 0.01, respectively (Student’s *t*-test).

To validate the regulatory relationship between circRNAs and mRNAs, we conducted qRT-PCR experiments. The predictions of miRNA and target gene interactions for the DE circRNAs have revealed numerous genes within the IR56 rice variety that are implicated in defense responses and plant protection (Supplementary Table S5). Further reinforcement for their significance in rice’s defense mechanisms is provided via GO and KEGG enrichments. From these predicted targets, we selected specific genes for validation through qPCR. These chosen targets displayed a negative correlation with the corresponding miRNA expression levels (Figure 6). Particularly in the IR-IR56-BPH group, we observed significant downregulation (log2 fold change > 2) in the circRNA (*novel_circ_0000490*). This circRNA appears to regulate the expression of the putative parental gene *Os12g06920* through its binding with *osa-miR166a-5p*.

Similarly, *novel_circ0000714* may interact with *osa-miR167a-5p* and *osa-miR167d-5p* and its putative parental genes *Os02g50330* and *Os11g10550* that encode NBS-LRR disease resistance proteins. Another circRNA, *novel_circ_0000189*, is linked to putative parental gene *Os01g48680*, contributing to cell death processes. Remarkably, significant upregulation in its transcript accumulation was observed in IR-IR56-BPH. Furthermore, the target of *novel_circ_0000536*, which corresponds to putative parental gene *Os01g24460*, responsible for encoding an R protein, exhibited a notable increase in expression in IR-IR56-BPH. Conversely, the miRNAs of downregulated circRNAs (*novel_circ_0000093* and *novel_circ_0000319*) in IR-IR56-BPH, including *osa-miR1850.3* and *osa-miR396c-3p*, demonstrated significant downregulated expression levels in comparison to the IR-CK group. However, the target putative parental genes *Os03g38330* and *Os01g06730*, associated with disease resistance, did not exhibit notable upregulated expression levels compared to the IR-CK. Similar observations were made in IR-TN1-BPH, where miRNAs of the upregulated circRNAs (*novel_circ_0000150* and *novel_circ_0000403*) were significantly downregulated. These miRNAs included *osa-miR396c-3p*, *osa-miR396a-5p*, *osa-miR396c-5p*, osa-*miR396e-5p*, and *osa-miR396g*. Notably, the predicted target genes (putative parental genes *Os11g24170*, *Os03g08900*, *Os12g10340*, and *Os11g40590*) encode NBS-LRR-type resistance or CC-NBS-LRR proteins.

**Figure 7:**
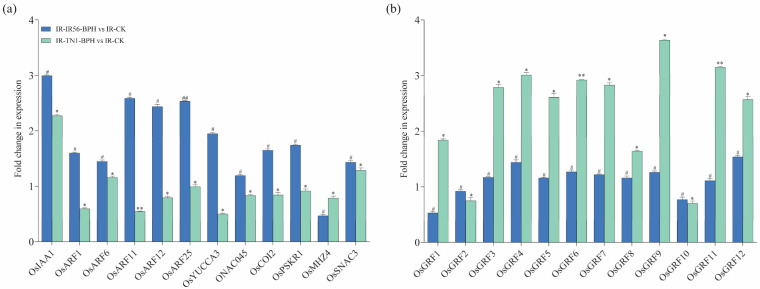
The qRT-PCR analysis of putative parental genes. (**a**) qRT–PCR analysis of the transcripts of some genes in pathways of phytohormones in rice. (**b**) Relative expression levels of the OsmiR396 target *OsGRF* genes in IR-IR56-BPH or IR-TN1-BPH as compared to the IR-CK group. Bars represent the mean ± SE of three biological replicates for the qPCR data. Bars represent the mean ± SE of three biological replicates for the qPCR data. Hashtags # and ## represent the significant difference in the expression levels of target genes in IR-IR56-BPH as compared to IR-CK, and asterisks * and ** indicate the significant difference in the expression levels of target genes in IR-TN1-BPH as compared to IR-CK at *p* < 0.05 and *p* < 0.01, respectively (Student’s *t*-test).

The potential involvement of mRNAs in rice phytohormone regulation, including JA, abscisic acid (ABA), ethylene (ET), cytokinin (CK), and auxin (IAA) is examined. qPCR experiments were conducted to confirm their respective expression levels (Figure 7a). Specifically, *OsIAA1* (*Os01g08320*) was predicted as a target of *osa-miR172d-5p*, upregulated in the IR-IR56-BPH group by *novel_circ_0000536*. *OsARF11* (Os04g56850) and *OsARF12* (*Os04g57610*) were predicted as targets of osa-*miR167a-5p*, upregulated in the IR-IR56-BPH group by *novel_circ_0000714*.

Conversely, the downregulated miR396 in IR-TN1-BPH demonstrated significant downregulation. miR396 is a conserved microRNA family crucial for plant growth and development. In rice, six genes, *OsmiR396a-f*, encode OsmiR396. Evidence suggests that OsmiR396a targets 12 growth-regulating factors (GRFs). This miRNA has demonstrated a negative regulatory effect on both biotic and abiotic stresses, as well as grain size. Transgenic rice plants sequestering OsmiR396 have shown increased grain size, enhanced seed quality, and improved resistance to BPH. To delve into the potential involvement of OsmiR396 in rice-BPH interactions (compatible or incompatible), we conducted qPCR to validate their relative expression (Figure 7b).

## 3. Discussion

### 3.1. Systematic Identification and Characterization Analysis of circRNAs Enhances Understanding of Two Brown Planthopper Populations with Varied Virulence Levels in Rice

CircRNAs, a unique class of non-coding RNAs, have garnered substantial attention due to their significant roles. A growing body of evidence underscores the vital functions of circRNAs in diverse biological processes, including stress responses, growth, and development [29]. The rapid progress in sequencing and bioinformatics technology has facilitated the identification of a plethora of circRNAs in various plant species, such as wheat [30], *Arabidopsis* [31], and maize [32], unveiling their pivotal contributions to distinct biological processes. Differential expression of circRNAs (DE circRNAs) has been observed during plant growth and development, and in response to biotic (pathogen or virus infection) and abiotic stresses (drought, salt, cold, heat, etc.) [29,33,34]. Some studies have explored the connection between circRNA expression and their parental genes, revealing significant correlations based on their expression levels across different samples in rice [34,35,36]. For instance, circR5g05160 has been verified to enhance rice immunity against *M. oryzae*, exemplifying the impact of circRNAs on rice–pathogen interactions. The diversity of circRNAs appears to influence different responses to *M. oryzae* infection in rice, unveiling a new layer of regulation in the rice–*M. oryzae* interaction [37].

However, as of now, there has been no systematic identification of circRNAs in the context of plant-BPH interactions. In this study, we initially identified a total of 186 circRNAs through high-throughput sequencing during infection by two BPH populations with different virulence levels. The number of circRNAs identified here was smaller compared to studies involving the developmental process of rice flag leaves from normal to senescence [35], as well as circRNAs implicated in the rice–*M. oryzae* interaction [37]. This variation in circRNA numbers likely stems from sample type and size, and potentially from the diversity of plant species. Notably, among the identified circRNAs, 39 were upregulated, and 43 were downregulated in IR-IR56-BPH vs. IR-CK (Figure 2a). Conversely, 42 miRNAs were upregulated in IR-TN1-BPH, while 42 miRNAs were downregulated (Figure 2b).

Categorization based on genomic location classifies circRNAs into exonic, intronic, and intergenic types. Exonic circRNAs constituted the majority (89.63%) among the identified 186 circRNAs (Figure 1b). This outcome underscores the intricate molecular mechanisms governing the biogenesis of circRNAs in plants. Chromosomal distribution analysis revealed the presence of circRNAs on every chromosome (Figure 1c), suggesting common features across rice circRNAs. This conservation implies potential shared biological functions among these circRNAs, warranting further investigation and validation.

### 3.2. circRNAs Involved in Defense against BPH Infestations in IR56 Rice Probably by Regulating Their Parental Genes

The GO enrichment analysis revealed similar biological processes and molecular functions among the targets of both IR-IR56-BPH and IR-TN1-BPH circRNAs. The KEGG pathway analysis displayed some common physiological processes for the target genes of circRNAs from IR-IR56-BPH and IR-TN1-BPH samples (Figure 3b). However, there were also distinct and exclusive KEGG enrichments observed for the targets of IR-IR56-BPH and IR-TN1-BPH. This suggests that enriched targets within different metabolic pathways may play roles in the response to BPH infestations in rice by regulating their protein-coding parental genes. Notably, these genes are closely associated with well-studied interactions, whether compatible or incompatible. Nevertheless, the roles of these circRNAs need to be experimentally validated using transgenic approaches.

Growing evidence in eukaryotic species highlights the potential of circRNAs to act as miRNA sponges, sequestering miRNAs away from their target mRNAs within circRNA–miRNA–mRNA regulatory networks. We discussed the potential involvement of these mRNAs in rice defense responses. In response to BPH infestation in IR56 rice, we observed the downregulation of *novel_circ_0000714* in both IR-IR56-BPH and IR-TN1-BPH samples. Predicted miRNAs, *osa-miR167a-5p* and *osa-miR167d-5p*, targeted parental genes *Os02g50330* and *Os11g10550*, which are associated with NBS-LRR disease resistance. Similarly, *novel_circ_0000454* showed downregulation in both IR-IR56-BPH and IR-TN1-BPH samples, with its parental gene, *Os04g28210*, encoding a leucine-rich repeat-containing protein, which has implications for disease resistance and brown planthopper resistance (https://browser.planteome.org/amigo/term/TO:0000424, accessed on 20 August 2023).

Likewise, *novel_circ_0000490* was downregulated in both IR-IR56-BPH and IR-TN1-BPH samples, with predicted *osa-miR166a-5p* targeting *Os12g06920*, encoding an NB-ARC domain-containing protein, and *Os02g51810*, encoding a putative mitochondrial outer membrane protein 64 (OsOM64). Notably, loss-of-function mutants of OM64 exhibited increased resistance to BPH [38]. Another example is *novel_circ_0000319*, downregulated in both IR-IR56-BPH and IR-TN1-BPH samples, predicted to be targeted by *osa-miR396c-3p*. Its parental gene contains leucine-rich repeat and N-terminal domains associated with disease resistance [39].

In the IR-IR56-BPH group, *novel_circ_0000093* was downregulated and predicted to be targeted by *osa-miR1850.3*. The target gene, *Os03g38330*, encodes an NB-ARC domain-containing protein. Similarly, *novel_circ_0000189* was downregulated in IR-IR56-BPH and targeted by *osa-miR1862f*, with its target gene, *Os01g48680* (two-pore calcium channel protein 1, *OsTPC1*), involved in regulating growth, development, and innate immune responses [40,41].

Furthermore, certain circRNAs, such as *novel_circ_0000074*, *novel_circ_0000576*, and *novel_circ_000086*, were upregulated in the IR-IR56-BPH group, potentially regulating miRNAs such as *osa-miR5788*, *osa-miR535-3p*, and *osa-miR397b*. Their target parental genes, including coiled-coil domain-containing protein 72 (*Os02g06450*), receptor-like cytoplasmic kinase 185 (*OsRLCK185*, *Os05g30870*), and LSD1-like-type zinc finger protein (disease resistance, *Os01g42710*), are known to be involved in rice defense responses [30,42,43,44].

These findings suggest that circRNAs associated with compatible interactions (IR56-BPH feeding on IR56 rice) may contribute to defense mechanisms, particularly through regulation of immune-related genes such as those encoding NB-ARC domain-containing proteins and receptor-like cytoplasmic kinases. Further investigation is required to confirm these roles through experimental validation.

### 3.3. circRNAs Involved in Phytohormones and Growth-Regulating Factors for BPH Infestations in IR56 Rice Probably by Regulating Their Parental Genes

Phytohormones are known to exert significant influence on diverse biological processes, including plant growth and development. Target genes implicated in various rice phytohormone pathways were observed in this study. Notably, genes associated with JA, ethylene, brassinosteroids (BRs), cytokinin, ABA, IAA, and ethylene pathways showed regulation during the compatible IR-IR56-BPH interaction. JAs have been recognized to mediate plant development and responses to environmental stresses [45]. In the IR-IR56-BPH group, an upregulated circRNA, *novel_circ_0000869*, corresponded to a parental gene called coronatine-insensitive protein (*COI2*, *Os03g0265500*), which encodes an F-box protein. This study revealed a positive correlation between the abundance of *novel_circ_0000869* and *Os03g0265500*, suggesting potential involvement of COI2 in downstream defense responses specific to COI2 and distinct from COI1 in BPH resistance [46].

Another instance in the IR-IR56-BPH group involves the upregulated *novel_circ_0000536*, whose parental gene is an F-box domain and kelch repeat-containing protein (*OsFBK12*, *Os03g0171600*). This protein interacts with *O. sativa* S-PHASE KINASE-ASSOCIATED PROTEIN1-LIKE PROTEIN (OsSAMS1), targeting OsSAMS1 for degradation and thereby affecting ethylene levels [47]. This coincides with the negative regulation of the ethylene signaling pathway in response to BPH infestation [48]. Similarly, the ethylene pathway was associated with *OsEREBP1* (*Os02g54160*), an AP2 domain-containing protein targeted by the upregulated *novel_circ_0000536* [49].

Brassinosteroids (BRs) are pivotal in plant growth, developmental processes, and pathogen responses. The parental gene glycogen synthase kinase (*OsGSK3*, *Os02g14130*) of the turn-point circRNA *novel_circ_0000189* was implicated in the negative regulation of BR signaling [50]. BRs have been shown to promote rice susceptibility to BPH by modulating the SA and JA pathways [51], suggesting that these pathways might be triggered during IR56-BPH feedings to undermine rice defenses.

In contrast, during the TN1-BPH (incompatible rice-BPH interaction), a multitude of cytokinin signaling-associated genes were identified, indicating roles in various abiotic stress stimuli. For instance, the downregulated *novel_circ_0000490* targeted a parental gene, A-type response regulator (*OsRR9*, *Os11g04720*), which is linked to stress–cytokinin interactions [52].

**Figure 8:**
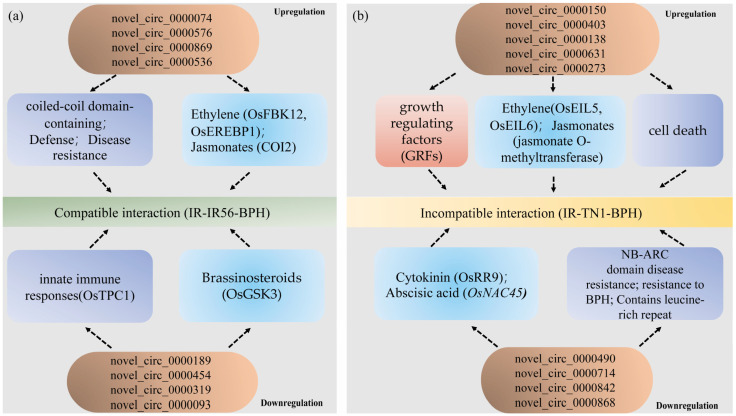
The proposed model of circRNAs regulating their parental genes to participate in the IR56 rice against the infestation of IR56-BPH and TN1-BPH. (**a**) The model of upregulated and downregulated circRNAs regulating mRNAs through the ceRNA network to participate in IR56-BPH infestations in the IR56 rice. (**b**) The model of upregulated and downregulated circRNAs regulating mRNAs through the ceRNA network to participate in TN1-BPH infestations in IR56 rice. The parental genes related to defense, phytohormone pathways, and growth-regulating factors are indicated by light purple, blue, and red. Arrows indicate a correlation.

Furthermore, during the upregulated IR-TN1-BPH group (incompatible interaction), genes encoding ethylene insensitive 3 domain-containing protein (*OsEIL5*, *Os02g36510*) and ethylene insensitive3-like 6 (*OsEIL6*, *Os04g57050*) were associated with jasmonate O-methyltransferase (*Os04g57050*), predicted targets of *osa-miR396a*/*c*/*e-5p*, and were involved in the rice-BPH interactions [23,27]. Moreover, the *GRF* genes, which encode growth-regulating factor proteins, were potentially regulated by novel_circ_0000150 and novel_circ_0000403 in the IR-TN1-BPH group, revealing a possible role of *OsmiR396* in mediating the compatible and incompatible interactions between rice varieties and BPH populations. In summary, the differentially expressed circRNAs identified in this study likely play pivotal roles in BPH infestations in IR56 rice by modulating defense regulatory networks, phytohormones, and growth-regulating factors (Figure 8). These findings offer insight into the complex interplay of circRNAs in response to BPH infestations and suggest their involvement in defense regulatory mechanisms.

## 4. Materials and Methods

### 4.1. Plant Materials and Growth Conditions

Two *indica* rice varieties IR56 and Taichung Native 1 (TN1) with contrasting BPH resistance were used. In a net house, pre-germinated IR56 rice seeds were planted in mud beds and grown under natural light and temperature conditions. The 14-day seedlings were kept in a temperature-controlled greenhouse at 28 ± 2 °C with 75–85% relative humidity and a photoperiod of 16 h of light and 8 h of dark in the China National Rice Research Institute (CNRRI).

### 4.2. Insect Materials and Growth Conditions

BPH colonies initially were collected from rice fields in Hangzhou, China and were maintained on TN1 rice (TN1-BPH) or IR56 rice (IR56-BPH) for more than 12 years at the CNRRI under the conditions described above [23]. TN1-BPH and IR56-BPH differ in their respective virulence levels [20].

### 4.3. BPH Bioassays and Sample Collections

Individual IR56 rice plants were infested with 4 newly emerged adult female BPHs and were sown in a plastic cage (10 cm in diameter, 60 cm in height) equipped with a net (with holes of diameter 0.5 mm); IR56 rice plants with no BPH treatment were put inside a plastic cage and served as IR-CK for this experiment, as previously descried [20,23]. At least three replicates were used for each BPH assay experiment. Plant stems (controls and treated) were collected after 24 h of BPH infestation and immediately frozen in liquid nitrogen. In order to prepare the samples for analysis, replicates of each sample were stored at −80 °C.

### 4.4. RNA Isolation and Detection

**Figure 9:**
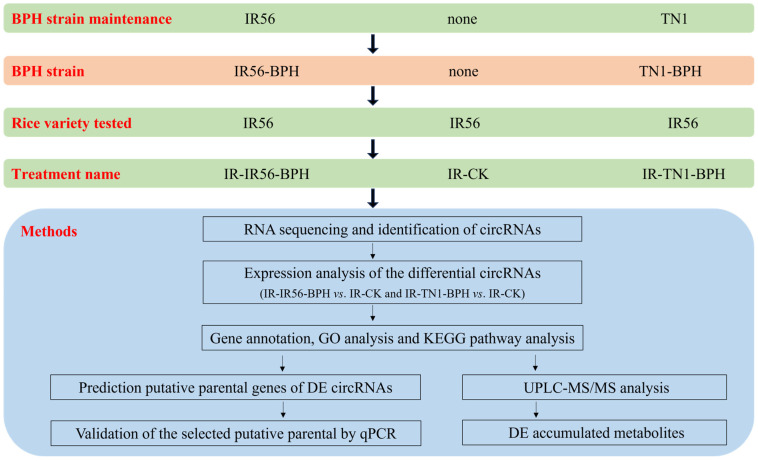
A schematic indicating the experimental design among the rice variety and brown planthopper (BPH) strain used in this study.

TransZol Up (Transgen, Beijing, China) was used to isolate total RNA from each sample (IR-CK, no BPH; IR56-BPH infested, IR-IR56-BPH; TN1-BPH infested, IR-TN1-BPH) (Figure 9). The isolation of total RNA was performed, as described by Nanda et al. [23]. Subsequently, the purity and concentration of total RNAs were detected using a NanoPhotometer spectrophotometer (Thermo-Fischer Scientific, Waltham, MA, USA). Then, the integrity of isolated RNA was evaluated using a Qubit RNA Assay Kit coupled with Qubit 2.0 Flurometer (Thermo-Fischer Scientific, Waltham, MA, USA) from Agilent 2100 (Thermo-Fischer Scientific, Waltham, USA). To ensure the application of qualified samples for sequencing, electrophoresis of 1% (*w*/*v*) agarose gels was used to monitor degradation and contamination of RNA. 

### 4.5. Library Construction and Circular RNA Sequencing

As described previously [36], circRNA libraries were constructed following the protocol after quality confirmation of total RNAs. The NEBNext Multiplex Circula RNA Library Prep Set for Illumina (New England Biolabs, Ipswich, MA, USA) was used to prepare an sRNA library for each sample using good quality RNA (>3 μg) using the manufacturer’s recommended method. The sequencing libraries were prepared using the NEBNext UltraTM Directional RNA Library Prep Kit for Illumina (Novegene, Beijing, China), according to the manufacturer’s instructions. Index codes were added to the sequencing libraries. Based on the manufacturer’s instructions, we clustered the index-coded samples using a cBot Cluster Generation System using TruSeq PE Cluster Kit v4-cBot-HS (Novegene). On an Illumina Hiseq 2500 platform, paired reads were generated from the resulting libraries following cluster generation. The sequencing data have been submitted to the NCBI’s GEO database (PRJNA1063489).

### 4.6. Identification of circRNAs

Before conducting genome-wide identification of circRNAs, raw reads were processed using in-house Perl scripts to filter out reads containing adapter sequences, poly-N tails, and low-quality reads. Subsequently, the Q20, Q30, and GC content was computed. The resulting clean reads were then aligned to the rice reference genome according to MSU-v7.0 (http://rice.plantbiology.msu.edu/, accessed on 10 July 2023) using Bowtie v1.2.3 [53]. The obtained reads that were unable to be mapped to the genomes were collected. In order to identify the unique anchor positions within these unmapped reads, the 20 nt anchors were initially extracted from both ends and independently aligned to the reference genomes of rice. The process of identification was conducted using the commonly utilized find_circ script (https://github.com/marvin-jens/find_circ, accessed on 19 July 2023) [54]. The altered orientation of the aligned anchors indicated the occurrence of circRNA splicing. Subsequently, the anchor alignments were prolonged in order to create the GT/AG splice sites surrounding the entire read alignments and breakpoints. Ultimately, a candidate circRNA was identified provided it possessed a minimum of two distinctly back-spliced reads. The identified circRNAs were produced along with annotation information. CircRNAs have been categorized into three types, including exonic, intronic, and intergenic circRNAs, based on their genomic origins. In order to ascertain and annotate protein-coding transcripts (mRNAs) from our transcriptome, the clean reads were aligned to the rice reference genome utilizing HISAT2 v2.1.0 [55]. Subsequently, the assembled reads were processed utilizing StringTie v1.3.5 [56].

### 4.7. Expression Analysis of the Differential circRNAs

To compare the expression of circRNAs across different virulence levels (IR-CK, IR-IR56-BPH, and IR-TN1-BPH), the back-spliced read accounts for each circRNA were standardized by utilizing the total number of sequencing reads in a corresponding sample dataset, which is defined as transcripts per million mapped reads (TPMs), as an indicator to determine their levels of expression. Python script (http://ccb.jhu.edu/software/stringtie/dl/prepDE.py, accessed on 22 July 2023) was used to obtain the transcript level raw read count matrix. The FPKMs value for protein-coding transcripts was calculated using StringTie [56] in order to determine the expression of these transcripts. A comparative examination of gene expression was conducted between two treatment groups (IR-IR56-BPH vs. IR-CK; IR-TN1-BPH vs. IR-CK) utilizing the DESeq v1.10.1 [57]. In this study, circRNAs or mRNA exhibiting adjusted *p*-value (*q* Value) and fold change > 1, as identified using DESeq, were considered to be differentially expressed. To explore whether circRNAs were conserved in different plant species, the back-splicing sequences of all identified circRNAs in this study were compared against the PlantcircBase database using BLAST v2023 [58].

### 4.8. Gene Annotation, GO Analysis, and KEGG Pathway Analysis of circRNAs

TargetFinder was used to predict the targets of the identified miRNA in *O. sativa* [59]. GO (Gene Ontology, http://www.geneontology.org/, accessed on 24 July 2023) and KEGG (Kyoto Encyclopedia of Genes and Genomes, http://www.genome.jp/kegg/, accessed on 25 July 2023), for enrichment analysis of the predicted target genes, were performed using the clusterProfiler R package [60].

### 4.9. Validation of the Selected DE circRNAs, miRNA, and mRNA Via Quantitative Real-Time PCR (qRT-PCR)

The circRNAs, miRNA, and mRNA candidates were validated using qRT-PCR. For reverse transcription (RT) of circRNA, we used an RT reagent kit purchased from GENESEED (Guangzhou, China). Real-time PCR was subsequently performed using SYBR Premix supplied by GENESEED (Guangzhou, China) using the Applied Biosystems 7500 Real-Time PCR Detection Systems (Applied Biosystems, Foster, CA, USA). Then, for miRNA, isolated total RNA was subjected to reverse transcriptase reactions with the TransScript Green miRNA Two-Step qRT-PCR SuperMix (Trans, Beijing, China) using the ABI 7500 real-time PCR system according to the manufacturer’s documentation. The validation of miRNA expression levels was subsequently performed using 2× PerfectStart Green qPCR SuperMix according to the manufacturer’s instructions. The ReverTra Ace qPCR RT Master Mix with gDNA Remover (Toyobo, Osaka, Japan) was used to perform reverse transcriptase reactions on total RNA. The validation of mRNA expression levels was subsequently performed using SYBR Green Realtime PCR Master mix kit (Toyobo, Osaka, Japan) with the ABI 7500 real-time PCR system through the implementation of quantitative real-time PCR (RT-qPCR) according to the manufacturer’s instructions.

The qRT-PCR data normalization was conducted using ubiquitin (*LOC_Os06g46770*), which served as an internal reference-gene control for the targets [23,61]. The relative RNA expression was evaluated using the 2^−ΔΔCt^ method [35,62]. To perform the qPCR analysis, three biological samples and three technical replicates of each biological sample were conducted. The primers for circRNAs were designed according to online resources such as CircPrimer 2.0, while the divergent primer was designed for qRT-PCR to amplify circRNAs using the “out-facing” strategy [63]. All primers for these genes were utilized using the National Center for Biotechnology Information (primer designing tool) and are shown in Supplementary Table S6.

### 4.10. Metabolomics and Data Analysis

Metabolomic analysis was conducted on individual samples representing different treatments: IR-CK, IR-IR56-BPH, and IR-TN1-BPH, as previously descried. Each treatment involved three biological replicates, with each replicate comprising 10 seedlings. The freeze-dried sample underwent grinding using a tissuelyser (64L, Jingxin, Shanghai, China) with a zirconia bead for 1 min at 50 Hz. The recovered extract solution (acetonitrile: methanol: water D 2:2:1, *v*/*v*) underwent vortexing for 30 s every 30 min, repeated five times in total, before being placed in a refrigerator at 4 °C overnight.

The samples were sonicated for 15 min in an ice bath, then centrifuged at 12,000 rpm for 15 min at 4 °C. The supernatant was collected, and the samples were vacuum dried. During mass spectrometry, 100 μL of an acetonitrile solution (acetonitrile: water = 1:1, *v*/*v*) was added for redissolving, followed by vortexing and centrifugation at 12,000 rpm for 10 min. The extracts were filtrated before UPLC-MS/MS analysis (Zhongkexinshengming, Shanghai, China). The UPLC column was Agilent, 1290 Infinity LC 1.8 µm (2.1 mm × 100 mm), with an injection volume of 2 μL; the flow rate was 0.5 mL/min; and the column temperature was 25 °C. A gradient elution was performed with water or acetonitrile containing 25 mM ammonium acetate and 25 mM ammonia liquor as aqueous and organic mobile phases, respectively. The samples were placed in a 4 °C automatic injector during the entire analysis process. An AB Triple TOF 6600 mass spectrometer (AB SCIEX, Framingham, MA, USA) was used to collect the primary and secondary spectra of the samples.

After separation using Agilent 1290 Infinity LC ultra-high performance liquid chromatography (UHPLC), the samples were analyzed using the Triple TOF 6600 mass spectrometer using positive and negative ion modes of electrospray ionization (ESI). ESI source parameters are as follows: the ion spray voltage of positive ions and negative ions was set to 5500 V, and a scan mode was adopted for mass spectrometry signal acquisition. 

A data evaluation based on the public database of metabolite information (https://massbank.eu/MassBank/, accessed on 25 September 2023) was performed. The primary and secondary spectral data of mass spectrometry were qualitatively analyzed using software Analyst 1.6.3 and quantitatively analyzed using MRM. The differentially accumulated metabolites (DAMs) were identified by variable importance in projection (VIP) ≥ 1 and fold change (FC) ≥ 2 or FC ≤ 0.5.

### 4.11. Statistical Analysis

Statistical analysis of the relative expressions was performed using Data Processing System software (v16.05). All values are presented as mean ± SE. Students’ *t*-tests were conducted using MS Excel and GraphPad Prism (v.8.0.1, GraphPad Software) to evaluate circRNA, miRNA expressions, and targets. In this study, statistical significance was defined as *p* values of 0.05 or 0.01.

## 5. Conclusions

In conclusion, our comparative analysis of circRNA profiles in IR56 rice exposed to virulent IR56-BPH and avirulent TN1-BPH populations highlights the functional significance of circRNAs. Differentially expressed circRNAs indicate BPH feeding induces circRNA transcription reconfiguration in IR56 rice. The similarity in DE miRNAs in both IR-IR56-BPH and IR-TN1-BPH interactions suggests a shared influence on target genes. Predictions of parental genes, enriched through GO and KEGG analyses, deepen our understanding of target functionality in rice-BPH interactions. This study introduces a conceptual framework for the regulatory network of IR56 rice in response to BPH attacks, marking the first exploration of circRNA roles in resistant rice plants. Further investigation, including overexpression, selective mutation, and MIMIC studies, is needed to strengthen circRNA–miRNA–target relationships’ credibility.

## Figures and Tables

**Table 1:** The display of internal ribosome entry site (IRES) identification.

CircRNA ID	Index	Score (>90%)
novel_circ_0000537_junction_seq	IRES	0.980183
novel_circ_0000433_junction_seq	IRES	0.963608
novel_circ_0000830_junction_seq	IRES	0.959345
novel_circ_0000371_junction_seq	IRES	0.955022
novel_circ_0000599_junction_seq	IRES	0.943738
novel_circ_0000521_junction_seq	IRES	0.942791
novel_circ_0000188_junction_seq	IRES	0.93967
novel_circ_0000177_junction_seq	IRES	0.938059
novel_circ_0000371	IRES	0.934103
novel_circ_0000446_junction_seq	IRES	0.932045
novel_circ_0000295_junction_seq	IRES	0.931279
novel_circ_0000737_junction_seq	IRES	0.929878
novel_circ_0000462_junction_seq	IRES	0.928313
novel_circ_0000454_junction_seq	IRES	0.921373
novel_circ_0000426_junction_seq	IRES	0.919793
novel_circ_0000294_junction_seq	IRES	0.91666
novel_circ_0000810_junction_seq	IRES	0.912211
novel_circ_0000319	IRES	0.908003

## Data Availability

Data are contained within the article and Supplementary Materials.

## Supplementary Materials

The supporting information can be downloaded at: https://www.mdpi.com/article/10.3390/plants13030373/s1, Figure S1. K-means clustering analysis and SOM clustering analysis; Table S1. Summary of circRNAs sequencing results in three rice samples; Table S2. Top 20 most abundant circRNAs expressed in the four libraries (TPM were shown); Table S3. List of the DE circRNA identified in IR56 rice; Table S4. Specific information regarding predicted miRNA and circRNA binding sites; Table S5. List of the predicted targets having a putative defense modulatory role in rice; Table S6. List of the primers used in qPCR for miRNA and target gene expression analysis.
