# Supplementary material for: Defense Regulatory Network Associated with circRNA in Rice in Response to Brown Planthopper Infestation
Source: Plants (Basel). 2024 Jan 26;13(3):373. doi: 10.3390/plants13030373 (PMC10857171; doi:10.3390/plants13030373)
Supplement: Supplementary file 1 [file plants-13-00373-s001.zip › plants-2759776-supplementary.pdf]

plants

Supplementary Figures and Tables

Figure S1. K-means clustering analysis and SOM clustering analysis.

Table S1. Summary of circRNAs sequencing results in three rice samples.

Table S2. Top 20 most abundant circRNAs expressed in the four libraries (TPM were shown).

Table S3. List of the DE circRNA identified in IR56 rice.

Table S4. Specific information regarding predicted miRNA and circRNA binding sites

Table S5. List of the predicted targets having a putative defense modulatory role in rice.

Table S6. List of the primers used in qPCR for miRNA and target gene expression analysis.

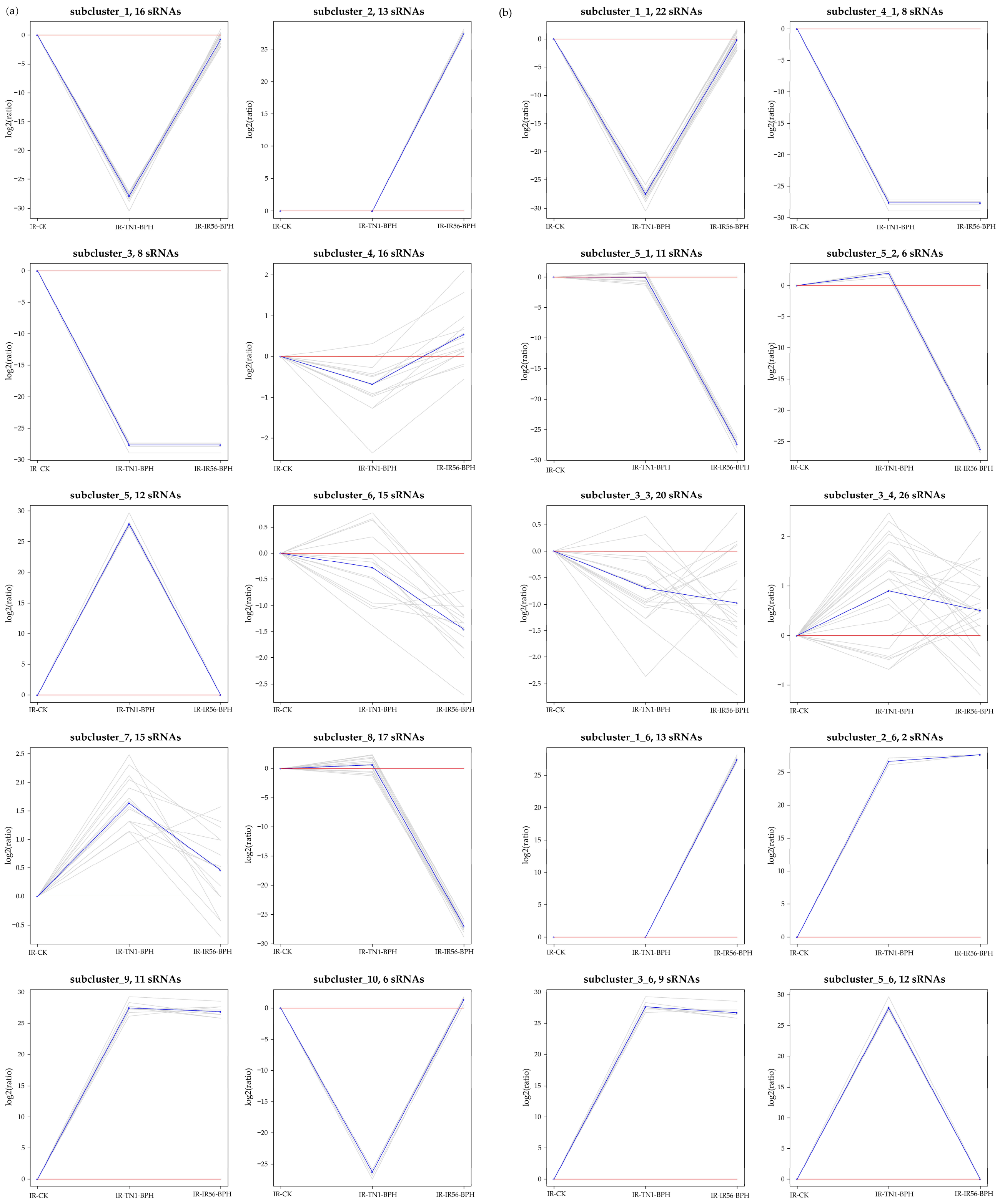

Figure S1. K-means clustering analysis and SOM clustering analysis. (a) K_means_clustering. (b) SOM clustering. Each subplot in the figure showcases a cluster of circRNA, where the grey lines represent the line graphs depicting the relative expression levels under different experimental conditions. The blue line signifies the trend within this particular

cluster. The gray lines in each subgraph represent line plots of the relative expression of circRNAs in a cluster under different experimental conditions. The blue line represents a line plot of the mean values of the relative expressions of all circRNAs in this cluster under different experimental conditions. Red line is for reference, online is up, offline is down. The X-axis represents the experimental conditions, and the Y-axis represents the relative expression.

Table S1. Summary of circRNAs sequencing results in three rice samples.

Sample	Raw counts	Clean Reads	Mapped reads(100%)	Uniquely mapped	Multiple mapped	Clean bases	Error_rate(%)	Q20(%)	Q30(%)	GC_content(%)
IR_CK	106174788	101459300	89434355 (88.15%）	76512867(75.41%)	12921488 (12.74%)	15.22 G	0.02	96.73	91.8	48.9
IR_IR56-BPH	95701674	91463396	80759198 （88.30%）	68008974(74.36%)	12750224 (13.94%)	13.72 G	0.02	96.7	91.75	47.93
IR_TN1-BPH	95646838	91019610	75616018 （83.08%）	65652190(72.13%)	9963828 (10.95%)	13.65 G	0.02	96.38	91.09	48.45

Table S2. Top 20 most abundant circRNAs expressed in the four libraries (TPM were shown).

circRNA.readcount	IR56_CK	circRNA.readcount	IR56_IR	circRNA.readcount	IR56_TN
novel_circ_0000588	311084.2646	novel_circ_0000588	344011.976	novel_circ_0000588	263888.8889
novel_circ_0000835	73995.77167	novel_circ_0000835	72155.68862	novel_circ_0000835	79954.95495
novel_circ_0000577	34128.66204	novel_circ_0000577	38922.15569	novel_circ_0000442	61186.18619
novel_circ_0000442	31410.45002	novel_circ_0000442	35329.34132	novel_circ_0000445	33408.40841
novel_circ_0000539	29598.30867	novel_circ_0000710	32335.32934	novel_circ_0000424	27402.4024
novel_circ_0000355	28994.26155	novel_circ_0000539	26047.90419	novel_circ_0000539	25900.9009
novel_circ_0000079	25672.00242	novel_circ_0000355	21856.28743	novel_circ_0000355	23273.27327
novel_circ_0000424	23557.83751	novel_circ_0000445	19760.47904	novel_circ_0000577	21396.3964
novel_circ_0000614	22349.74328	novel_circ_0000414	19461.07784	novel_circ_0000079	16141.14114
novel_circ_0000414	19027.4 8414	novel_circ_0000614	19461.07784	novel_circ_0000614	15390.39039
novel_circ_0000445	16913.31924	novel_circ_0000424	17065.86826	novel_circ_0000414	14639.63964
novel_circ_0000710	15101.17789	novel_circ_0000444	17065.86826	novel_circ_0000444	14639.63964
novel_circ_0000842	13289.03654	novel_circ_0000079	15868.26347	novel_circ_0000065	10510.51051
novel_circ_0000444	12987.01299	novel_circ_0000842	11676.64671	novel_circ_0000803	10510.51051
novel_circ_0000065	10872.84808	novel_circ_0000065	8682.634731	novel_circ_0000029	9384.384384
novel_circ_0000803	10268.80097	novel_circ_0000803	8682.634731	novel_circ_0000210	9384.384384
novel_circ_0000415	9664.753851	novel_circ_0000210	8383.233533	novel_circ_0000571	9384.384384
novel_circ_0000210	7248.565388	novel_circ_0000472	7185.628743	novel_circ_0000413	8633.633634
novel_circ_0000571	6946.54183	novel_circ_0000029	6586.826347	novel_circ_0000627	8633.633634
novel_circ_0000177	6342.494715	novel_circ_0000415	6586.826347	novel_circ_0000133	8258.258258

Table S3. List of the DE circRNA identified in IR56 rice.

ID	IR56_IR	IR56_CK	log2.Fold_change.	p.value	q.value
novel_circ_0000503	0	4905.958	-13.26	0	0
novel_circ_0000559	940.0277	3463.029	-1.8813	0	0
novel_circ_0000746	0	4617.373	-13.173	0	0
novel_circ_0000771	626.6852	3751.615	-2.5817	0	0
novel_circ_0000783	940.0277	3463.029	-1.8813	0	0
novel_circ_0000675	0	2597.272	-12.343	3.28E-295	3.37E-295
novel_circ_0000189	1253.37	3463.029	-1.4662	1.31E-280	1.25E-280
novel_circ_0000714	0	2308.686	-12.173	5.64E-271	4.43E-271
novel_circ_0000134	0	2020.101	-11.98	8.46E-246	4.92E-246
novel_circ_0000541	0	2020.101	-11.98	8.46E-246	4.92E-246
novel_circ_0000870	0	2020.101	-11.98	8.46E-246	4.92E-246
novel_circ_0000133	2193.398	4617.373	-1.0739	1.65E-244	9.19E-245
novel_circ_0000093	1253.37	3174.444	-1.3407	1.77E-228	8.73E-229
novel_circ_0000490	1253.37	3174.444	-1.3407	1.77E-228	8.73E-229
novel_circ_0000120	0	1731.515	-11.758	1.63E-219	6.06E-220
novel_circ_0000408	0	1731.515	-11.758	1.63E-219	6.06E-220
novel_circ_0000521	0	1731.515	-11.758	1.63E-219	6.06E-220
novel_circ_0000048	626.6852	2020.101	-1.6886	4.01E-197	1.31E-197
novel_circ_0000308	626.6852	2020.101	-1.6886	4.01E-197	1.31E-197
novel_circ_0000433	626.6852	2020.101	-1.6886	4.01E-197	1.31E-197
novel_circ_0000142	0	1442.929	-11.495	6.95E-192	1.66E-192
novel_circ_0000339	0	1442.929	-11.495	6.95E-192	1.66E-192
novel_circ_0000454	0	1442.929	-11.495	6.95E-192	1.66E-192
novel_circ_0000518	0	1442.929	-11.495	6.95E-192	1.66E-192
novel_circ_0000658	0	1442.929	-11.495	6.95E-192	1.66E-192
novel_circ_0000696	0	1442.929	-11.495	6.95E-192	1.66E-192
novel_circ_0000827	0	1442.929	-11.495	6.95E-192	1.66E-192
novel_circ_0000483	1253.37	2885.858	-1.2032	1.75E-179	4.02E-180
novel_circ_0000855	0	1154.343	-11.173	1.43E-162	3.18E-163

novel_circ_0000892	0	1154.343	-11.173	1.43E-162	3.18E-163
novel_circ_0000128	940.0277	2308.686	-1.2963	2.86E-159	6.26E-160
novel_circ_0000463	1253.37	2597.272	-1.0512	3.37E-134	6.72E-135
novel_circ_0000165	0	865.7574	-10.758	4.88E-131	9.05E-132
novel_circ_0000305	0	865.7574	-10.758	4.88E-131	9.05E-132
novel_circ_0000643	0	865.7574	-10.758	4.88E-131	9.05E-132
novel_circ_0000677	940.0277	2020.101	-1.1037	4.46E-112	7.84E-113
novel_circ_0000063	0	577.1716	-10.173	2.48E-96	3.99E-97
novel_circ_0000673	0	577.1716	-10.173	2.48E-96	3.99E-97
novel_circ_0000770	0	577.1716	-10.173	2.48E-96	3.99E-97
novel_circ_0000319	626.6852	1442.929	-1.2032	1.20E-90	1.78E-91
novel_circ_0000373	626.6852	1442.929	-1.2032	1.20E-90	1.78E-91
novel_circ_0000496	626.6852	1442.929	-1.2032	1.20E-90	1.78E-91
novel_circ_0000605	626.6852	1442.929	-1.2032	1.20E-90	1.78E-91
novel_circ_0000480	3133.426	0	12.614	0	0
novel_circ_0000575	4073.454	865.7574	2.2342	0	0
novel_circ_0000710	33841	14429.29	1.2298	0	0
novel_circ_0000889	4073.454	0	12.992	0	0
novel_circ_0000151	5013.481	1731.515	1.5338	6.65E-306	7.40E-306
novel_circ_0000813	2506.741	0	12.292	9.37E-276	8.34E-276
novel_circ_0000536	3760.111	1154.343	1.7037	6.61E-272	5.52E-272
novel_circ_0000080	2193.398	0	12.099	4.88E-250	3.26E-250
novel_circ_0000625	2193.398	0	12.099	4.88E-250	3.26E-250
novel_circ_0000830	2193.398	0	12.099	4.88E-250	3.26E-250
novel_circ_0000071	1880.055	0	11.877	3.39E-223	1.37E-223
novel_circ_0000317	1880.055	0	11.877	3.39E-223	1.37E-223
novel_circ_0000462	1880.055	0	11.877	3.39E-223	1.37E-223
novel_circ_0000582	1880.055	0	11.877	3.39E-223	1.37E-223
novel_circ_0000766	1880.055	0	11.877	3.39E-223	1.37E-223
novel_circ_0000576	2820.083	865.7574	1.7037	2.35E-204	8.28E-205
novel_circ_0000069	1566.713	0	11.614	5.42E-195	1.48E-195
novel_circ_0000103	1566.713	0	11.614	5.42E-195	1.48E-195
novel_circ_0000152	1566.713	0	11.614	5.42E-195	1.48E-195
novel_circ_0000213	1566.713	0	11.614	5.42E-195	1.48E-195
novel_circ_0000335	1566.713	0	11.614	5.42E-195	1.48E-195
novel_circ_0000461	1566.713	0	11.614	5.42E-195	1.48E-195
novel_circ_0000749	1566.713	0	11.614	5.42E-195	1.48E-195
novel_circ_0000869	1566.713	0	11.614	5.42E-195	1.48E-195
novel_circ_0000389	2193.398	577.1716	1.9261	9.11E-192	2.13E-192
novel_circ_0000371	1880.055	577.1716	1.7037	7.56E-137	1.55E-137
novel_circ_0000801	1880.055	577.1716	1.7037	7.56E-137	1.55E-137
novel_circ_0000074	940.0277	0	10.877	6.27E-133	1.21E-133
novel_circ_0000271	940.0277	0	10.877	6.27E-133	1.21E-133
novel_circ_0000810	2193.398	865.7574	1.3411	1.23E-107	2.14E-108
novel_circ_0000223	626.6852	0	10.292	1.43E-97	2.39E-98
novel_circ_0000270	626.6852	0	10.292	1.43E-97	2.39E-98
novel_circ_0000820	626.6852	0	10.292	1.43E-97	2.39E-98
novel_circ_0000768	2506.741	1154.343	1.1187	7.02E-89	1.03E-89
novel_circ_0000028	1566.713	577.1716	1.4407	2.47E-87	3.51E-88
novel_circ_0000522	1566.713	577.1716	1.4407	2.47E-87	3.51E-88
novel_circ_0000602	1566.713	577.1716	1.4407	2.47E-87	3.51E-88
novel_circ_0000560	1880.055	865.7574	1.1187	4.21E-67	5.41E-68
novel_circ_0000590	1253.37	577.1716	1.1187	2.57E-45	2.96E-46

**ID**	**IR56_TN**	**IR56_CK**	**log2.Fold_change.**	**p.value**	**q.value**

novel_circ_0000365	0	5070.749	-13.308	0	0
novel_circ_0000415	1788.646	10141.5	-2.5033	0	0
novel_circ_0000490	0	3486.14	-12.767	0	0
novel_circ_0000503	0	5387.671	-13.395	0	0
novel_circ_0000559	0	3803.062	-12.893	0	0
novel_circ_0000710	0	15846.09	-14.952	0	0
novel_circ_0000842	6439.126	13944.56	-1.1148	0	0
novel_circ_0000868	0	2852.297	-12.478	1.11E-302	5.69E-303
novel_circ_0000089	0	2535.375	-12.308	9.59E-278	3.62E-278
novel_circ_0000356	0	2535.375	-12.308	9.59E-278	3.62E-278
novel_circ_0000714	0	2535.375	-12.308	9.59E-278	3.62E-278
novel_circ_0000760	0	2535.375	-12.308	9.59E-278	3.62E-278
novel_circ_0000007	0	2218.453	-12.115	7.58E-252	2.12E-252
novel_circ_0000303	0	2218.453	-12.115	7.58E-252	2.12E-252
novel_circ_0000308	0	2218.453	-12.115	7.58E-252	2.12E-252
novel_circ_0000541	0	2218.453	-12.115	7.58E-252	2.12E-252
novel_circ_0000870	0	2218.453	-12.115	7.58E-252	2.12E-252
novel_circ_0000771	1430.917	4119.984	-1.5257	8.36E-247	2.28E-247
novel_circ_0000120	0	1901.531	-11.893	8.15E-225	1.80E-225
novel_circ_0000151	0	1901.531	-11.893	8.15E-225	1.80E-225
novel_circ_0000537	0	1901.531	-11.893	8.15E-225	1.80E-225
novel_circ_0000142	0	1584.609	-11.63	2.06E-196	3.73E-197
novel_circ_0000202	0	1584.609	-11.63	2.06E-196	3.73E-197
novel_circ_0000373	0	1584.609	-11.63	2.06E-196	3.73E-197
novel_circ_0000496	0	1584.609	-11.63	2.06E-196	3.73E-197
novel_circ_0000518	0	1584.609	-11.63	2.06E-196	3.73E-197
novel_circ_0000605	0	1584.609	-11.63	2.06E-196	3.73E-197
novel_circ_0000696	0	1584.609	-11.63	2.06E-196	3.73E-197
novel_circ_0000797	0	1267.687	-11.308	2.73E-166	4.44E-167
novel_circ_0000243	1788.646	4119.984	-1.2038	1.89E-163	3.03E-164
novel_circ_0000828	1073.188	2852.297	-1.4102	3.57E-150	5.58E-151

novel_circ_0000295	2146.375	4436.906	-1.0477	1.02E-135	1.56E-136
novel_circ_0000094	1788.646	3803.062	-1.0883	2.75E-125	3.88E-126
novel_circ_0000561	1788.646	3803.062	-1.0883	2.75E-125	3.88E-126
novel_circ_0000242	715.4585	1901.531	-1.4102	1.02E-100	1.38E-101
novel_circ_0000408	715.4585	1901.531	-1.4102	1.02E-100	1.38E-101
novel_circ_0000028	0	633.8437	-10.308	2.71E-98	3.48E-99
novel_circ_0000389	0	633.8437	-10.308	2.71E-98	3.48E-99
novel_circ_0000522	0	633.8437	-10.308	2.71E-98	3.48E-99
novel_circ_0000801	0	633.8437	-10.308	2.71E-98	3.48E-99
novel_circ_0000319	715.4585	1584.609	-1.1472	1.27E-58	1.41E-59
novel_circ_0000454	715.4585	1584.609	-1.1472	1.27E-58	1.41E-59
novel_circ_0000063	2861.834	633.8437	2.1747	0	0
novel_circ_0000180	6439.126	1267.687	2.3447	0	0
novel_circ_0000271	3219.563	0	12.653	0	0
novel_circ_0000273	2861.834	0	12.483	0	0
novel_circ_0000413	8227.772	0	14.006	0	0
novel_circ_0000426	4292.751	1584.609	1.4378	0	0
novel_circ_0000590	2861.834	633.8437	2.1747	0	0
novel_circ_0000810	3577.292	950.7655	1.9117	0	0
novel_circ_0000889	6081.397	0	13.57	0	0
novel_circ_0000890	6439.126	2218.453	1.5373	0	0
novel_circ_0000892	5365.938	1267.687	2.0816	0	0
novel_circ_0000325	2504.105	633.8437	1.9821	9.18E-299	4.53E-299
novel_circ_0000101	2504.105	0	12.29	7.25E-288	3.21E-288
novel_circ_0000138	2504.105	0	12.29	7.25E-288	3.21E-288
novel_circ_0000631	5723.668	2852.297	1.0048	1.54E-279	6.59E-280
novel_circ_0000305	2861.834	950.7655	1.5898	3.13E-260	1.12E-260
novel_circ_0000899	2861.834	950.7655	1.5898	3.13E-260	1.12E-260
novel_circ_0000108	2146.375	0	12.068	2.73E-257	8.55E-258
novel_circ_0000150	2146.375	0	12.068	2.73E-257	8.55E-258
novel_circ_0000230	2146.375	0	12.068	2.73E-257	8.55E-258
novel_circ_0000403	2146.375	0	12.068	2.73E-257	8.55E-258
novel_circ_0000820	2146.375	0	12.068	2.73E-257	8.55E-258

novel_circ_0000074	1788.646	0	11.805	3.98E-225	9.28E-226
novel_circ_0000078	1788.646	0	11.805	3.98E-225	9.28E-226
novel_circ_0000174	1788.646	0	11.805	3.98E-225	9.28E-226
novel_circ_0000188	1788.646	0	11.805	3.98E-225	9.28E-226
novel_circ_0000223	1788.646	0	11.805	3.98E-225	9.28E-226
novel_circ_0000239	1788.646	0	11.805	3.98E-225	9.28E-226
novel_circ_0000270	1788.646	0	11.805	3.98E-225	9.28E-226
novel_circ_0000602	2146.375	633.8437	1.7597	3.60E-222	7.57E-223
novel_circ_0000673	2146.375	633.8437	1.7597	3.60E-222	7.57E-223
novel_circ_0000770	2146.375	633.8437	1.7597	3.60E-222	7.57E-223
novel_circ_0000737	3577.292	1584.609	1.1747	2.04E-217	4.16E-218
novel_circ_0000205	2504.105	950.7655	1.3971	1.00E-192	1.79E-193
novel_circ_0000335	1430.917	0	11.483	5.69E-191	9.74E-192
novel_circ_0000749	1430.917	0	11.483	5.69E-191	9.74E-192
novel_circ_0000830	1430.917	0	11.483	5.69E-191	9.74E-192
novel_circ_0000286	2861.834	1267.687	1.1747	2.88E-174	4.87E-175
novel_circ_0000103	1073.188	0	11.068	3.49E-154	5.53E-155
novel_circ_0000165	2146.375	950.7655	1.1747	3.82E-131	5.63E-132
novel_circ_0000560	2146.375	950.7655	1.1747	3.82E-131	5.63E-132
novel_circ_0000625	715.4585	0	10.483	1.27E-113	1.75E-114

Table S4. Specific information regarding predicted miRNA and circRNA binding sites.

miRNA_ID	target_circRNA	Target_aligned_fragment	match	miRNA_aligned_fragment
osa-miR396c-3p	novel_circ_0000319	CUCUUCAAAGCUUUCU-GACG	:: ..:: ::::::: :::	GAAGGGUGUCGAAA-GAACUGG
osa-miR166i-3p	novel_circ_0000305_junction_seq	GGUGACCUGAA-GCCUGGUUCGG	:. :: ::::::::.:.::.	CUCCUU-ACUUCGGAC-UAGGCU
osa-miR166e-5p	novel_circ_0000490	CCUUGA-CGAGAUAAAAUUCC	:::::: : :::.:: :::::	GGAACUUGGUCUGUUGU-AAGG
osa-miR5837.2	novel_circ_0000114	UGCUUAAUG-UCCACAUAACC	:::. ::.: :::::: :::	ACGGCUUGCGAGGUG-UAGUGG
osa-miR6249a	novel_circ_0000065	GUCUCUGGCGAGCUCUAC-GCC	:.: :.:::::::: :.:	CGGCGGCCGCUCGAGAA-GUGC
osa-miR439i	novel_circ_0000604	UCGAGCUGCCGCG-GAUCUACA	::::.: .::::: :: :::	AGCUUGUUGGCGCCAA-GCUGU
osa-miR2102-5p	novel_circ_0000871	GCGGCGGCGGCGGCG-GCCG	: ::::::::::: :::	CACCGCCGCCGCCGAACGGG
osa-miR5535	novel_circ_0000734	AUGCGGGGUUGAAUCA-GCAGA	::::.:. :: :::: :::::	UACGUCUGAAGUUAGUG-CGUCU
osa-miR396b-5p	novel_circ_0000403	UAAUUGUAAGA-GAGCUGUUGAA	.: :: .:::.::::: :::	GUCAA-GUUCUUUCGACAC-CUU
osa-miR395t	novel_circ_0000453	GCAUUUCCCCAA-CGCUUCAA	: :::::::: :.:::::	CUCAAAGGGGUUUGUGAA-GUG
osa-miR396g	novel_circ_0000150	CAGUACAGGAG-GAGCCUGUGGG	: :: ::.::. ::::::::.	GGCAAGUUCUU-UCG-GACACCU
osa-miR1862g	novel_circ_0000189	CCUAAACAAACCACCUUCAA	:: ::: ::::: :.:::	GGUUUUAUUUGGUUGGA-GUA
osa-miR535-3p	novel_circ_0000576	AGUG-CAACGGGAGGGUGCAG	:::: :::::::::.. :::	UCACUGUUGCCCU-CUUUCGUG
osa-miR439a	novel_circ_0000604	UCGAGCUGCCGCG-GAUCUACA	::::.: .::::: :: :::	AGCUUGUUGGCGCCAA-GCUGU
osa-miR396d	novel_circ_0000150	CAGUACAGGAG-GAGCCUGUGGG	: :: ::.::. ::::::::.	GGCAAGUUCUU-UCG-GACACCU
osa-miR11337-3p	novel_circ_0000627	CGAGGACGAGUGUAAC-GAGGUC	:::: ::::.: :::::::.:	GCUC-UGCUUAGAUUGCU-CUAU
osa-miR166l-3p	novel_circ_0000305	GUGACCUGAA-GCCUGGUUCGG	. : :::::::::::.::.	UCCCUAACUUCGGAC-CAGGCU
osa-miR1848	novel_circ_0000503	UACGUGCGCGCGGCGUG-CGAGG	: :..:::::: :: ::::::	ACGUGCGCGCGCGGC-CGCUCC
osa-miR439c	novel_circ_0000604	UCGAGCUGCCGCG-GAUCUACA	::::.: .::::: :: :::	AGCUUGUUGGCGCCAA-GCUGU
osa-miR5079a	novel_circ_0000295	AUGCCCACAUAGCAGAUG-CAAG	::.:: : :::.:::: :::.	UAUGGUUUUAUUGUCUAGGUUU
osa-miR5079a	novel_circ_0000462	AUUCUGGGCUAACAGA-CCAAA	:: :.... :::::: :::::	UAUGGUUUUAUUGUCUAGGUUU
osa-miR5075	novel_circ_0000604	GUCGGCGGCGGCGAC-GAAGGC	:. :.:::::::::: ::.	CGCCUGCCGCCGCUGCCU-CUU
osa-miR5075	novel_circ_0000007	GCGGAUGGUG-GCGAGGGACAG	:::::.::.:::: ::: :.	CGCCUGCCGCCGCUGCCU-CUU
osa-miR439h	novel_circ_0000604	UCGAGCUGCCGCG-GAUCUACA	::::.: .::::: :: :::	AGCUUGUUGGCGCCAA-GCUGU
osa-miR5079b	novel_circ_0000295	AUGCCCACAUAGCAGAUG-CAAG	::.:: : :::.:::: :::.	UAUGGUUUUAUUGUCUAGGUUU
osa-miR5079b	novel_circ_0000462	AUUCUGGGCUAACAGA-CCAAA	:: :.... :::::: :::::	UAUGGUUUUAUUGUCUAGGUUU

osa-miR166a-5p	novel_circ_0000490	CCUUGA-CGAGAUAAAAUUCC	:::::: : :::.:: :::::	GGAACUUGGUCUGUUGU-AAGG
osa-miR439d	novel_circ_0000604	UCGAGCUGCCGCG-GAUCUACA	::::.: .:::::: :: :::	AGCUUGUUGGCGCCAA-GCUGU
osa-miR2122	novel_circ_0000005	GAGCAAGAGGUUAUUUC-GAGA	::.:::.::::::::: ::.:	CUUGUUUUCCAAUAAAAAC-UUU
osa-miR5539a	novel_circ_0000229	CGGCGCGCAC-GUCCGUCUUCUU	.::.::: :.:::: :::::	AUCGUGCGCGUAGGCAAAA-GAA
osa-miR5788	novel_circ_0000074	UGCUCGGGGAU-GUUACAAUCCA	:.:: :.: ::::.::: ::::	AUGAUCUCAUACAGUGU-AGGU
osa-miR396a-5p	novel_circ_0000403	UAAUUGUAAGA-GAGCUGUUGAA	.: :: .::::.:::::: :::	GUCAA-GUUCUUUCGACAC-CUU
osa-miR2093-3p	novel_circ_0000658	AUCCAUCAAAUGGACA-GAUGU	:: ::: :: :::: ::::::	UACGUAAUUAACCU-UCUACA
osa-miR2905	novel_circ_0000760	UAC-CUUUUUUACUGAUAUGCA	: ::::: :.::::.::: :	ACGGAAACAGUGACUGUA-CAU
osa-miR6249b	novel_circ_0000065	GUCUCUGGCGAGCUCUAC-GCC	:.: :.:::::::::: :.:	CGGCGGCCGCUCGAGAA-GUGC
osa-miR166m	novel_circ_0000305	GUGACCUGAA-GCCUGGUUCGG	. : ::::::::::.::.	UCCCUUACUUCGGAC-CAGGCU
osa-miR2931	novel_circ_0000582	UUCUGAAAUCAGUAAGAAAG	:: ::: ::::..:: ::::	AAAACUGUAGUUGUUAUUUC
osa-miR810a	novel_circ_0000539	UCGCAUUUGGUGGGCU-AUAA	.:.::: :::::::: :: :	GGUGUACAC-CACCCGAAUACU
osa-miR2876-3p	novel_circ_0000093	GCAU-UGGUAGUUCAUAUGUGAA	::: ..:: ::::::::. :::	CGUUGUCA-CAA-GUAUAUCCUU
osa-miR439e	novel_circ_0000604	UCGAGCUGCCGCG-GAUCUACA	::::.: .:::::: :: :::	AGCUUGUUGGCGCCAA-GCUGU
osa-miR1862f	novel_circ_0000189	CCUAAACAAACCACCUUCAA	:: ::: :::::: :.:::	GGUUUUAUUUGGUUGGA-GUA
osa-miR5539b	novel_circ_0000229	CGGCGCGCAC-GUCCGUCUUCUU	.::.::: :.:::: :::::	AUCGUGCGCGUAGGCAAAA-GAA
osa-miR439f	novel_circ_0000604	UCGAGCUGCCGCG-GAUCUACA	::::.: .:::::: :: :::	AGCUUGUUGGCGCCAA-GCUGU
osa-miR11342-3p	novel_circ_0000627	UCUUCGAGGACGAGUGUAAC-GAG	: : :::: ::::.: :::::::	AAACGCUC-UGCUUAGAU-UGCUC
osa-miR1440a	novel_circ_0000781	AGGAAAUUGG-AGUUGAGCA	:::: : ::: : :::::::	UCCUCUCACCAUAAACUCGU
osa-miR5081	novel_circ_0000371	GCUAUUAAUUUGC-ACU-AAUUC	.::::.::::::: :: ::::	UGAUAGUUAAACGAUGUUU-AAU
osa-miR413	novel_circ_0000766	GUGCAGCAACAGGUGGAAC-CAC	:::::: ::::.:::.::: :	CACGUC-UUGUUCACU-UUGAUC
osa-miR5809	novel_circ_0000286	GACGUGG-CGCCGGUGAUGA	: :::: ::::::.::.::	CGACACCAGCGGCCGCUGCU
osa-miR5809	novel_circ_0000604	GCAGUCGGCGGCGGCGACGA	:: :: : :: :::::::::	CGACACCAGCGGCCGCUGCU
osa-miR5077	novel_circ_0000007	AGGUCAACCUGGCGGCGAAC	::: ::::.:.:: :::::	ACCACUUGGGCUGC-GCUUG
osa-miR2871b-3p	novel_circ_0000424	GUGACAA-GGAAGCUGAAAUC	::::: : .:::.::.::::	CACUGGUAUCUUUGAU-UUUAU
osa-miR171i-3p	novel_circ_0000313	UAUGUUGGCG-GGCUUGAUCC	::.:::.:: ::::..::::	CUAUAACUGCGCCGAGUUAGG
osa-miR171i-3p	novel_circ_0000313_junction_seq	UAUGUUGGCG-GGCUUGAUCC	::.:::.:: ::::..::::	CUAUAACUGCGCCGAGUUAGG
osa-miR166k-3p	novel_circ_0000305	GUGACCUGAA-GCCUGGUUCGG	. : ::::::::::.::.	UCCCUAACUUCGGAC-CAGGCU
osa-miR439g	novel_circ_0000604	UCGAGCUGCCGCG-GAUCUACA	::::.: .:::::: :: :::	AGCUUGUUGGCGCCAA-GCUGU
osa-miR2924	novel_circ_0000308	GAGGCGGAC-GAGGCAGGCGAG	: ::::: :: .:::.:::::	CAC-CGCCGGCCUCGUUCGCUC
osa-miR390-3p	novel_circ_0000065	GGAGGAUCAGGA-GAGGUAGUG	:::: :::::: ::.:::.:	CCUCG-AGUCCUAUCUAUCGC

osa-miR2871a-3p	novel_circ_0000424	GUGACAA-GGAAGCUGAAAUC	::::: : .:::.::.::::	CACUGGUAUCUUUGAU-UUUAU
osa-miR439b	novel_circ_0000604	UCGAGCUGCCGCG-GAUCUACA	::::.: .:::::: :: :::	AGCUUGUUGGCGCCAA-GCUGU
osa-miR1850.3	novel_circ_0000093	AAGAGUGUGGUGAACUA-GAUAA	:::: :: ::::::::.:.:	UUCUAACUACACUUGAU-UUGUC
osa-miR396h	novel_circ_0000150	CAGUACAGGAG-GAGCCUGUGGG	: :: ::.::. :::::::.	GGCAAGUUCUU-UCG-GACACCU
osa-miR172d-5p	novel_circ_0000536	CUGAA-CGUGGUGGUGCUGG	:::: : ::.::::::::	CACUUAGAACUACCACGACG
osa-miR166j-3p	novel_circ_0000305	GUGACCUGAA-GCCUGGUUCGG	: : :::::::::::.::.	CCCCUUACUUCGGAC-CAGGCU
osa-miR2118d	novel_circ_0000065	UAGUUGAGGGAGG-AUCAG-GAG	::: .. :::::: :::::::.	AUCCGUACCCUCCGUAGUCCUU
osa-miR5522	novel_circ_0000869	AU-CCUGUAGUUCCUAUUGUU	:: ::: . .::::::::::	UACGGAGGGUAAGGAU-AACAA
osa-miR2926	novel_circ_0000229	GGCGC-GGCGUCGACUACCU	.::.: ..::::::: ::::	UCGUGGUUGCAGCUGCUGGA
osa-miR166f	novel_circ_0000305	GUGACCUGAA-GCCUGGUUCGG	: : :::::::::::.::.	CCCCUUACUUCGGAC-CAGGCU
osa-miR166b-3p	novel_circ_0000305	GUGACCUGAA-GCCUGGUUCGG	: : :::::::::::.::.	CCCCUUACUUCGGAC-CAGGCU
osa-miR166d-3p	novel_circ_0000305	GUGACCUGAA-GCCUGGUUCGG	: : :::::::::::.::.	CCCCUUACUUCGGAC-CAGGCU
osa-miR166a-3p	novel_circ_0000305	GUGACCUGAA-GCCUGGUUCGG	: : :::::::::::.::.	CCCCUUACUUCGGAC-CAGGCU
osa-miR166h-3p	novel_circ_0000305	GUGACCUGAA-GCCUGGUUCGG	: : :::::::::::.::.	CUCCUUACUUCGGAC-CAGGCU
osa-miR396c-5p	novel_circ_0000403	AAUUGUAAGA-GAGCUGUUGAA	:: : .::::.:::::: :::	UUCAAGUUCUUUCGACAC-CUU
osa-miR166g-3p	novel_circ_0000305	GUGACCUGAA-GCCUGGUUCGG	: : :::::::::::.::.	CUCCUUACUUCGGAC-CAGGCU
osa-miR6248	novel_circ_0000130	UGUUAUCCUCAAUCUUCAAAUG	:..:: :::: :::.:::::.	AUGAU-GGAGGUAG-GAGUUUAU
osa-miR2118o	novel_circ_0000065	UAGUUGAGGGAGG-AUCAG-GAG	::: . :::::: :::::::	AUCCGAACCCUCCGUAGUCCUC
osa-miR397b	novel_circ_0000869	AAUGGAA-GCUGCCCUCAAUAA	:: .: ::::: ::::::::	GUAGUUGCGACGUG-AGUUAUU
osa-miR435	novel_circ_0000294	AAAACAUCAAUACCGGAUUA	::: .:::::::::: :	AGUUGAGGUUAUGGCCUAUU
osa-miR166c-5p	novel_circ_0000449	CUCAAACCAGGCAAUGUUCU	::: :::::.:::..:::.	GAGCCUGGUCUGUUGUAAGG
osa-miR2928	novel_circ_0000696	CUGCAAAAUCUC-UCUUCUU	: .:::::: :: :::::::	GUUGUUUUACAGCAGAAGAA
osa-miR166c-3p	novel_circ_0000305	GUGACCUGAA-GCCUGGUUCGG	: : :::::::::::.::.	CCCCUUACUUCGGAC-CAGGCU
osa-miR3982-5p	novel_circ_0000426	AUUGUUGUCUGCGUG-GAACAC	::::::::.::.:::::: : :	UAACAACGGAUGCAC-CUCGCG
osa-miR5152-5p	novel_circ_0000539	AGAAAUC-UGCUUAUUUCUAU	::: ::: :::.:::..:::.	UCU-CUAGUACGGAUAGGAUG
osa-miR2925	novel_circ_0000007	CCGCAGCCUGUGGUCGCCG	:: ::::.:.::.::::.	UGCUUCGGGCGCCGGCGGU
osa-miR2925	novel_circ_0000257	CCGGCGCCCGCGGCCGCGU	::. :::::::::::	UGCUUCGGGCGCCGGCGGU
osa-miR166l-5p	novel_circ_0000454	UCUUGAACAUAGGCAACAGUCG	.::::::: .::.:::::.::	GGAACUUG-GUCUGUUGUUAGG
osa-miR167b	novel_circ_0000714	CUGCUCGUGCUCGG-CAGCUUCA	: ::.:::: :::::::::	AUCUAGUACGA-CCGUCGAAGU
osa-miR167a-5p	novel_circ_0000714	CUGCUCGUGCUCGG-CAGCUUCA	: ::.:::: :::::::::	AUCUAGUACGA-CCGUCGAAGU
osa-miR5339	novel_circ_0000078	UCAGAGAUGGAGUCU-CUAUCUA	:: :::: .:: ::::::::	AGACUCU-UCUAAGAGAUA-GAC
osa-miR167c-5p	novel_circ_0000714	CUGCUCGUGCUCGG-CAGCUUCA	: ::.:::: :::::::::	AUCUAGUACGA-CCGUCGAAGU

osa-miR2093-5p	novel_circ_0000490	UGUA-UUUGAAUUAAUGCAC	::: ::. :::::::::::	ACAAGAAGGUUAAUUACGUG
osa-miR2926	novel_circ_0000577	GUCGCCAACGUCGGCGACUC	. :.:::::::::.::::.	UCGUGGUUGCAGCUGCUGGA
osa-miR166h-3p	novel_circ_0000305_junction_seq	GGUGACCUGAA-GCCUGGUUCGG	:. :: :::::::::::.::.	CUCCUU-ACUUCGGAC-CAGGCU
osa-miR396e-5p	novel_circ_0000150	CAGUACAGGAG-GAGCCUGUGGG	:::: ::.::. :::::::::.	GUCAAGUUCUU-UCG-GACACCU
osa-miR166g-3p	novel_circ_0000305_junction_seq	GGUGACCUGAA-GCCUGGUUCGG	:. :: :::::::::::.::.	CUCCUU-ACUUCGGAC-CAGGCU
osa-miR166l-3p	novel_circ_0000305_junction_seq	GGUGACCUGAA-GCCUGGUUCGG	.: :: :::::::::::.::.	UCCCUA-ACUUCGGAC-CAGGCU
osa-miR5075	novel_circ_0000750	GCGGUCGCUGGCGACGGG-GAU	:::: :: .::::::::.::	CGCCUGCCGCCGCUGCCU-CUU
osa-miR5536	novel_circ_0000768	CGGUCAUGGUGGUCACUAC-CAUU	: ..:::..:: ::::::::::	GAUGGUAUUAC-AGUGAUGGUAA
osa-miR2925	novel_circ_0000210	ACGCGGUCCGCCGCCGCCG	::: .:.:::: ::::::.	UGCUUCGGGCGCCGGCGGU
osa-miR1436	novel_circ_0000414	AUUAC-CUUUGUCCCAAAAUGU	:.: :::..:::::: :::::	UGAGGGAGGCAGGGUAUUACA
osa-miR166k-3p	novel_circ_0000305_junction_seq	GGUGACCUGAA-GCCUGGUUCGG	.: :: :::::::::::.::.	UCCCUA-ACUUCGGAC-CAGGCU
osa-miR166j-3p	novel_circ_0000305_junction_seq	GGUGACCUGAA-GCCUGGUUCGG	:: :: :::::::::::.::.	CCCCUU-ACUUCGGAC-CAGGCU
osa-miR167g	novel_circ_0000714	CUGCUCGUGCUCGG-CAGCUUCA	: : ::.:::: :::::::::	GUCUAGUACGA-CCGUCGAAGU
osa-miR167e-5p	novel_circ_0000714	CUGCUCGUGCUCGG-CAGCUUCA	: : ::.:::: :::::::::	GUCUAGUACGA-CCGUCGAAGU
osa-miR166f	novel_circ_0000305_junction_seq	GGUGACCUGAA-GCCUGGUUCGG	:: :: :::::::::::.::.	CCCCUU-ACUUCGGAC-CAGGCU
osa-miR166b-3p	novel_circ_0000305_junction_seq	GGUGACCUGAA-GCCUGGUUCGG	:: :: :::::::::::.::.	CCCCUU-ACUUCGGAC-CAGGCU
osa-miR166d-3p	novel_circ_0000305_junction_seq	GGUGACCUGAA-GCCUGGUUCGG	:: :: :::::::::::.::.	CCCCUU-ACUUCGGAC-CAGGCU
osa-miR166a-3p	novel_circ_0000305_junction_seq	GGUGACCUGAA-GCCUGGUUCGG	:: :: :::::::::::.::.	CCCCUU-ACUUCGGAC-CAGGCU
osa-miR6256	novel_circ_0000750	UACGCCUACAAC-GACUAC-UAC	:::.:::::::: :: ::::::	AUGUGGAUGUUGGCUCAU-GAUG
osa-miR167d-5p	novel_circ_0000714	CUGCUCGUGCUCGG-CAGCUUCA	: : ::.:::: :::::::::	GUCUAGUACGA-CCGUCGAAGU
osa-miR167f	novel_circ_0000714	CUGCUCGUGCUCGG-CAGCUUCA	: : ::.:::: :::::::::	GUCUAGUACGA-CCGUCGAAGU
osa-miR166c-3p	novel_circ_0000305_junction_seq	GGUGACCUGAA-GCCUGGUUCGG	:: :: :::::::::::.::.	CCCCUU-ACUUCGGAC-CAGGCU
osa-miR2925	novel_circ_0000007	UCGACGCCCGCGCCCGCCG	::: ::::::: :::::.	UGCUUCGGGCGCCGGCGGU
osa-miR167h-5p	novel_circ_0000714	CUGCUCGUGCUCGG-CAGCUUCA	: : ::.:::: :::::::::	GUCUAGUACGA-CCGUCGAAGU
osa-miR1440a	novel_circ_0000243	UGGGGAGUGGUAAUUGAGCC	::.:::::::: ::::::	UCCUCUCACCAUAAACUCGU
osa-miR167i-5p	novel_circ_0000714	CUGCUCGUGCUCGG-CAGCUUCA	: : ::.:::: :::::::::	GUCUAGUACGA-CCGUCGAAGU
osa-miR166e-3p	novel_circ_0000305	GUGACCUGAA-GCCUGGUUCGG	: : ::::::::::::::.	CCCCUUACUUCGGACCAA-GCU
osa-miR5809	novel_circ_0000007	CCUGUGGUCGCCGGCGGCCG	:::::::::::::::.: .	CGACACCAGCGGCCGCUGCU
osa-miR5809	novel_circ_0000750	GAGGCGGUCGCUGGCGACGG	: : :::::.:::::::.	CGACACCAGCGGCCGCUGCU
osa-miR167j	novel_circ_0000714	CUGCUCGUGCUCGG-CAGCUUCA	: : ::.:::: :::::::::	GUCUAGUACGA-CCGUCGAAGU
osa-miR5499	novel_circ_0000433	GUCCA-ACUGAUUCUUCCUUC	:::: : .:::::::::::	AAGGUAUUGCU-AAGAAGGAAG
osa-miR5075	novel_circ_0000210	GCCAACGGCGGCGGCG-GAGGA	:: :::::::::.:::::.:	CGCCUGCCGCCGCUGCCU-CUU

osa-miR5499	novel_circ_0000319	UUUC-UGACGAUUCUUUCUUU	::.: :.:::::::::.:::.	AAGGUAUUGCU-AAGAAGGAAG
osa-miR5075	novel_circ_0000007	GCGG-CGGCGGCGGCGGGGAG	:::: ::::::::.:::.::.	CGCCUGCCGCCGCUGCCU-CUU
osa-miR166e-3p	novel_circ_0000305_junction_seq	GGUGACCUGAA-GCCUGGUUCGG	:: :: ::::::::::::::.	CCCCUU-ACUUCGGACCAA-GCU

Table S5. List of the predicted targets having a putative defense modulatory role in rice.

circRNA	miRNA	Putative mRNA	Putative function
**IR-IR56 vs IR56-CK**			
novel_circ_0000490	osa-miR166a-5p	Os12g06920	NBS-LRR disease resistance
novel_circ_0000454	osa-miR166l-5p	Os04g28210	disease resistance
novel_circ_0000305	osa-miR166m	Os04g49890	multidrug resistance-associated
novel_circ_0000714	osa-miR167a-5p	Os02g50330	resistance
novel_circ_0000714	osa-miR167d-5p	Os11g10550	NBS-LRR disease resistance
novel_circ_0000093	osa-miR1850.3	Os03g38330	disease resistance
novel_circ_0000189	osa-miR1862f	Os01g48680	cell death
novel_circ_0000319	osa-miR396c-3p	Os01g06730	disease resistance
novel_circ_0000536	osa-miR172d-5p	Os01g24460	R protein
novel_circ_0000074	osa-miR5788	Os02g06450	coiled-coil domain-containing protein
novel_circ_0000576	osa-miR535-3p	Os05g30870	defense
novel_circ_0000869	osa-miR397b	Os01g42710	disease resistance
circRNA	miRNA	Putative mRNA	Putative function
**IR-TN1 vs IR56-CK**			
novel_circ_0000490	osa-miR166a-5p	Os02g51810	resistance to SSB
novel_circ_0000319	osa-miR396c-3p	Os11g24170	CC-NBS-LRR protein,
novel_circ_0000454	osa-miR166l-5p	Os01g69080	disease resistance
novel_circ_0000714	osa-miR167a-5p	Os12g29290	disease resistance
novel_circ_0000490	osa-miR166a-5p	Os11g31500	Durable Resistance to Rice stripe viru
novel_circ_0000714	osa-miR167d-5p	Os01g67580	multidrug resistance-associated protein
novel_circ_0000403	osa-miR396a-5p	Os03g08900	disease resistance
novel_circ_0000403	osa-miR396c-5p	Os03g08900	disease resistance
novel_circ_0000150	osa-miR396e-5p	Os11g40590	cell death
novel_circ_0000074	osa-miR5788	Os02g06450	coiled-coil domain-containing
novel_circ_0000150	osa-miR396g	Os12g10340	NBS-LRR type resistance

Table S6. List of the primers used in qPCR for miRNA and target gene expression analysis.

Genes	Forward primer (5′-3′)	Reverse primer (5′-3′)
novel_circ_0000503	TTACTACCAGCACCGTGTCG	GGTGGCCAGCTCAACCTTAT
novel_circ_0000714	GCAGGATAGGCCATCTGGAG	GTAGCTCAGCATCCGTGGAC
novel_circ_0000305	GGCCATCTGGAGTTCTGACG	CCGTGATCTTCACGTCCGAA
novel_circ_0000063	AGGACCGAACGCCAATTTGA	TCGTTCACCTCCACGTATCC
novel_circ_0000270	ACAAAATTCCTGGTCCGGCA	GCATCAACACTTTGGAGAGTACA
novel_circ_0000074	AACGATCGATCCAGCGTCAC	ATCCCCGAGCATCACACTTC
novel_circ_0000271	CAGTTCCGGACACCCAATCC	TTCAGGAATCCCTTCTGGCTG
novel_circ_0000830	GTGGATCAACAGCAGTCACG	CAGCATCTCGTGTCCCCATT
novel_circ_0000480	CCGCGTCGGATTTTACTGC	AGTACACTCTCGGTCTTGGC
novel_circ_0000889	AAATGGTGGATGACCCCGCT	CCCTGGATACATCACACCGA
novel_circ_0000559	TCTCTCGGCTCCGATCTCAA	CCCTCAGGTGCCATCCATC
novel_circ_0000490	CAAGGAGAGGTTGGAGGTCT	CACCGCTTTGCGTAGGCTAT
novel_circ_0000868	ATTGACCGGACGGCATACAC	TGCGTCATCTATCCTTCTCGC
novel_circ_0000089	CTGCATGTTGTTTGAAGACCCA	CCCATCCACTTCAGGCCTTC
novel_circ_0000522	GCAATGTGTCCCAATGATGGTT	CCAGTCCAAAGCTCAATAGGC
novel_circ_0000801	AAGGTCCAAACCCGCTCTTC	TTCTCCCTCGACCCAGATGT
novel_circ_0000625	TGTTGTACGTGTCATTGATGCT	AGTGCACGTCCAGAAAATGC
novel_circ_0000103	TGTCAGATGGGGACGTTGAC	CCTGCCATGCTTCCATTTGT
novel_circ_0000335	ACCTTACCAGCTTCTTGCAGT	GTAGAGCTTTGGCTTGGGCT
novel_circ_0000749	GTGATGATCGGGGGTGTCAT	TTCCCGCAAACTAGCAGCAT
novel_circ_0000101	AGAGTTGGTCAACCTGAGTGTC	GCTCAGGGTATGGAGTGGAC
novel_circ_0000138	CCTTCCTCCCTCCCAAAGAG	ATTCTGACGGCAGGAAACGC
novel_circ_0000273	CAGTTCCGGACACCCAATCC	TTCAGGAATCCCTTCTGGCTG
novel_circ_0000093	ACATGAGGTTGTGCGGATCG	GGAGAAACATATGCAGCGAGC
novel_circ_0000189	GGCATTGGCCAACACAATCA	ACGCAGATGCAGCCTATGTT
novel_circ_0000536	CTCCCACACCTCACCATGTC	AGCAGCCGAGCGATTATTGA
novel_circ_0000576	GCCGGACAGGGAGGGCCC	TGCACCCCGGGCTCGTCG
novel_circ_0000869	CCACAGATTGTCTTGAATGAT	TTTTGCCGAACGGCTAATTGAT
novel_circ_0000319	TTGAGGGTTGTGAACCCTCTTCA	CTGTTAATGGTATAATGCCCTCT
novel_circ_0000454	GAAAAATCGTCAGGTCACCTGGAG	AGAAGTTGAGTTTGCTTCTGT
novel_circ_0000403	ACCCTGACATCAACGACTTCCAACT	AATTACATTGTTGGTTGTTATCT
novel_circ_0000150	CGGTGCTTCTGGACTCGGCGAGCAA	AGACCGAGGCCCTTGGCGGA
qmiR166a-5p	CGCGGGAATGTTGTCTGGT	
qmiR166l-5p	CGCGGGATTGTTGTCTGGT	
qmiR166m	GCGTCGGACCAGGCTTCA	
qmiR167a-5p	GCGTGAAGCTGCCAGCAT	
qmiR167d-5p	GCGTGAAGCTGCCAGCAT	
qmiR1850.3	GCGCGCTGTTTAGTTCACATC	
qmiR1862f	GCGCGATGAGGTTGGTTTA	
qmiR396c-3p	CGCGGGTCAAGAAAGCTGT	
qmiR172d-5p	GCGGCAGCACCATCAAG	
qmiR5788	GCGCGTGGATGTGACATACT	
qmiR535-3p	GCGGTGCTTTCTCCCGTT	
qmiR397b	CGCGTTATTGAGTGCAGC	
qmiR396g	CGCGTCCACAGGCTTTCTT	
qmiRNA-R	AGTGCAGGGTCCGAGGTATT	
miRU6	TACAGATAAGATTAGCATGGCCCC	GGACCATTTCTCGATTTGTACGTG

qPCR-LOC_Os12g06920	GAGATTGGTGCACTGGGTCA	AGGCCATTTACCGGCATCAA
qPCR-LOC_Os04g28210	GGCTCGCCTACAATCTCCTC	ATGAGAAATCGGGCAGCACA
qPCR-LOC_Os03g38330	GTCAACCTCCGCCATCTCAA	TGGTGTAGCTCGGCAACATT
qPCR-LOC_Os01g48680	TTGCAAGTTCGGCGTTTCAG	ATCTGCAAACCAAGGGAGCA
qPCR-LOC_Os01g06730	ATTGGGCATGGGAGAACTGG	GGAAGTGGTATTGGCCCCTC
qPCR-LOC_Os01g24460	TTGGTTGTAGTAGCCCTGCG	AAATGGCCAGAGGGAGCAAT
qPCR-LOC_Os05g30870	GCAATGTGTTTGCAGGAGCA	ATTGCTCCGACTGTGCTGAA
qPCR-LOC_Os01g42710	ATGCTTTGGCAGCAACAGTG	GGCGCTTCTTGCATTATGGG
qPCR-LOC_Os02g51810	CTGCCAACCACAGCTGGATA	TGACAGCAGCCTGAAAGTGT
qPCR-LOC_Os11g24170	TAGAGCGGATGTGTGAAGCC	CACCTTTGGCTCAGTGGAGT
qPCR-LOC_Os01g69080	ACTCTCGATGGTGTCCGAGA	CATGCCTGTTCTCCTCAGCA
qPCR-LOC_Os12g29290	GGGTGGGCTGAATAGTCTCG	CTCCTCGAATTGTGGGCAGT
qPCR-LOC_Os01g67580	CATATGGCGGAGCTCTCGTT	ACTTGTTCCTGCCTCTCAGC
qPCR-LOC_Os03g08900	GCACCTACTACCTCATCGGC	GAATTAGCGTCTGCACTGCG
qPCR-LOC_Os11g40590	GTGTTGGCAAAGCTGAGTCG	CCTTTGGAATTGCAGGTGGC
qPCR-LOC_Os02g06450	AGGCCGCCAAGAAGGATTAC	CACTTTTCTTGAGACCGGCG
Os02g53690 (OsGRF1)	AAAGAGGACGACGATGAGAAAGAG	GCCCAGGAGGAAGCAGTG
Os06g10310 (OsGRF2)	TACGGACGGCAAGAAGTG	GGCATTTCCACAGGCTTTC
Os04g51190 (OsGRF3)	CAATGCTGCGTCTTACTC	AATGTGGAGGTCTGAGAAG
Os02g47280(OsGRF4)	CATCTGTTGTCGGTTCTG	GCAATAGCAGGGTAAAGAG
Os06g02560 (OsGRF5)	TTCTTCTCAGGAGCATCAG	GTTCAAGGTGGGAGTAGG
Os03g51970 (OsGRF6)	CCTCGCTATCAACCATCAG	GCACTTGTTCACTCTCATTATG
Os12g29980 (OsGRF7)	TTGGATCAGGTGGCTATC	TTGTGTTGGTGTGAATGG
Os11g35030 (OsGRF8)	GCAAGAGCAAGAGCAAGATG	GCAAGAGCAAGAGCAAGATG
Os03g47140 (OsGRF9)	GCTCATTGCCATCTTCTGTC	GTTCGCCATTGTCCTGTTC
Os02g45570 (OsGRF10)	TGCTCATCTACCGCTACTTC	CGACGCTCTTCCAGATGG
Os04g24190 (OsGRF11)	TGCCTACTCATCTCGTCTTC	GTTCTGGTTCTGGGTTCTTTC
Os04g48510(OsGRF12)	TCAAGAAAGCCTATGGAAGCCTCTG	TGGCGACGGTGTGGAGTG
qOs04g57610-OsARF12	CCCGGATATCGGTTGGGATG	CCCATATACCTGCGAACGCT
qOs03g15880-OsCOI2	TTGTGCGCAATTGTCGAAGG	CAAAGGAACCACCGGCAAAG
qOs02g41890-OsPSKR1	ATCCGTGTTCTCCGGTTCAC	TTGTGCAGTTCCCAAACCCT
qOs01g03750-MHZ4	GCCGTCCTCCCTTTCTACAC	GTCGGGAGTCCAGGATAGGT
qOs01g13520-OsARF1	TGGATGGAGCACGTTTGTGA	CTGTTTCTGCACAAGACGCC
qOs01g08320-OsIAA1	GACGTACGAGGACAAGGACG	TTTCATGAGACGAAGGCGCT
qOs11g03370-ONAC045	AGCCACCAAGGAGGGATACT	CTCCATAGCTGGTAGGCTGC
qOs04g56850-OsARF11	CTACGACCCCATGAGATGGC	CAAGGTCGACGAAGGGAACA
qOs01g09550-SNAC3	AAGCTTGACAGGAGGTCTGC	GGCTGGTAAGGCCATTCTGT
qOs02g06910-OsARF6	ACTACTCCGGGCTGCTTAGA	TTCACAAAGGTTGCATGCGG
qOOs12g41950-OsARF25	CTAGGGCAGGTGCAGTTTCA	GCAGCTGCTGAGTGTTATGC
qOs01g53200-OsYUCCA3	GAGCTTCCCCTCATGCCTTT	ATCCCAAACCTTGCAGCGTA
OsUbiquitin	CCAGTAAGTCCTCAGCCATGGAG	GGACACAATGATTAGGGATC
